# Allogenic adipose-derived stem cell therapy overcomes ischemia-induced microvessel rarefaction in the myocardium: systems biology study

**DOI:** 10.1186/s13287-017-0509-2

**Published:** 2017-03-09

**Authors:** Gemma Vilahur, Blanca Oñate, Judit Cubedo, Maria Teresa Béjar, Gemma Arderiu, Esther Peña, Laura Casaní, Manuel Gutiérrez, Antoni Capdevila, Guillem Pons-Lladó, Francesc Carreras, Alberto Hidalgo, Lina Badimon

**Affiliations:** 1 Cardiovascular Research Center (CSIC-ICCC) Hospital de la Santa Creu i Sant Pau (HSCSP), c/Sant Antoni Ma Claret 167, 08025 Barcelona, Spain; 2 CIBERCV, ISCIII, Madrid, Spain; 3 Radiology Unit (HSCSP), Barcelona, Spain; 4 Cardiology Unit (HSCSP), Barcelona, Spain; 5 Cardiovascular Research Chair, UAB (Autonomous University of Barcelona), Barcelona, Spain

**Keywords:** Allogenic adipose-derived stem cells, Conditioned media, Angiogenesis, Myocardial infarction, Proteomics, Systems biology

## Abstract

**Background:**

Myocardial microvascular loss after myocardial infarction (MI) remains a therapeutic challenge. Autologous stem cell therapy was considered as an alternative; however, it has shown modest benefits due to the impairing effects of cardiovascular risk factors on stem cells. Allogenic adipose-derived stem cells (ASCs) may overcome such limitations, and because of their low immunogenicity and paracrine potential may be good candidates for cell therapy. In the present study we investigated the effects of allogenic ASCs and their released products on cardiac rarefaction post MI.

**Methods:**

Pig subcutaneous adipose tissue ASCs were isolated, expanded and GFP-labeled. ASC angiogenic function was assessed by the in-vivo chick chorioallantoic membrane (CAM) model. Pigs underwent MI induction and 7 days after were randomized to receive: allogenic ASCs (intracoronary infusion); conditioned media (CM; intravenous infusion); ASCs + CM; or PBS/placebo (control). Cardiac damage and function were monitored by 3-T cardiac magnetic resonance imaging upon infusion (baseline CMR) and 1 and 3 weeks thereafter. We assessed in the myocardium: microvessel density; angiogenic markers (CD105, CD31, TF, VEGFR2, VEGFR1, vWF, eNOS, CD62); collagen deposition; and reparative fibrosis (TGFβ/TβRII/collagen). Differential proteomics of ASCs and CM was performed to characterize the ASC protein signature.

**Results:**

CAM indicated a significant ASC proangiogenic capacity. In pigs after MI, only PBS/placebo animals displayed an impaired cardiac function 3 weeks after infusion (*p* < 0.05 vs baseline). Administration of ASCs + CM significantly enhanced neovessel formation and favored cardiac repair post MI (*p* < 0.05 vs the other groups). Molecular markers of angiogenesis were significantly upregulated both at transcriptional and protein levels (*p* < 0.05). The in-silico bioinformatics analysis of the ASC and CM proteome (interactome) indicated activation of a coordinated protein network involved in the formation of microvessels and the resolution of rarefaction.

**Conclusion:**

Coadministration of allogenic ASCs and their CM synergistically contribute to the neovascularization of the infarcted myocardium through a coordinated upregulation of the proangiogenic protein interactome.

**Electronic supplementary material:**

The online version of this article (doi:10.1186/s13287-017-0509-2) contains supplementary material, which is available to authorized users.

## Background

Advances in pharmacological therapy and interventional techniques have increased the rate of survival in patients suffering from acute myocardial infarction (MI) [1]. Yet the development of heart failure at a later time point due to reduced perfusion, microvessel rarefaction, irreversible cardiomyocyte loss and consequent adverse cardiac remodeling has increased dramatically [2]. Implantation of adult stem cells into the ischemic damaged myocardium has been investigated for its potential to repair/regenerate the injured cells within the infarct zone [3]. However, recent systematic review and meta-analysis have evidenced that administration of autologous stem cells exerts modest and limited benefits in ischemic heart disease (IHD) patients [4]. The presence of cardiovascular risk factors and metabolic disorders in cardiac patients seems to negatively influence the effects of adult stem/progenitor stem cells, discouraging their autologous use in the clinical setting [5]. In this regard, allogenic adipose-derived stem cells (ASCs) from obese patients display an impaired angiogenic potential [6, 7] and clustering of risk factors has been shown to reduce ASC and bone-marrow-derived cell pluripotency and self-renewal [8–11]. The use of allogenic stem cells from healthy donors may overcome these limitations.

In contrast to other adult stem cells, mesenchymal stem cells (MSCs) have been demonstrated to have immunomodulatory and immunosuppressive properties [12]. Moreover, MSCs are considered to be immunoprivileged due to lack of expression of class II major histocompatibility complex and costimulatory molecules on the cell surface [13] and, consequently, allogeneic administration of MSCs does not stimulate donor-specific alloimmune reactions as shown in several clinical trials [14, 15]. Emerging evidence supports that among all MSCs those derived from the adipose tissue (i.e., ASCs) hold great potential for allogenic use because, in addition to their inherent and proven low immune reactivity [16, 17], they are highly abundant, easily accessible and expandable in culture. Administration of ASCs has so far shown promising results across a wide range of therapeutic applications including bone–cartilage defects, inflammatory-based disease and IHD [18]. In this latter regard, several animal studies seem to indicate that administration of ASCs exerts cardiac beneficial effects as compared with the administration of placebo-control media likely through paracrine/autocrine-related mechanisms [16, 19]. Many experimental studies concur that most of the benefits associated with the administration of ASCs are largely mediated by the actions of cytokines and growth factors secreted by the ASCs rather than by ASC transdifferentiation and engraftment [20–23]. Because microvessel rarefaction is a common pathogenic landmark in the ischemic myocardium, here we sought to determine in a pig model of MI using clinical standard perioperative procedures (antiplatelet treatment) and operating blindly [24] whether coadministration of ASCs and conditioned media (CM) could overcome myocardial rarefaction and whether the effects were due to ASCs or their secretome.

## Methods

Materials and methods are expanded in Additional file 1.

### Ethical approval

The experimental procedures with animals were reviewed and approved by the Institutional Animal Care and Use Committees (CEEA-ICCC) and authorized by the Animal Experimental Committee of the local government (# 5601) in accordance with Spanish law (RD 53/2013) and European Directive 2010/63/EU.

### Experimental design

Pigs (*n* = 20) were subjected to MI induction. One week post MI, the animals were randomized to receive: ASCs (1 × 10^7^ cells); CM (30 ml); ASCs and their CM; or PBS (control; 30 ml). ASC coronary delivery was performed with an over-the-wire catheter and total balloon occlusion for 2 min. CM was administered intravenously. Cell preparations were administered blindly by the surgical operators. Animals were then brought to the cardiac magnetic resonance imaging (CMR) facilities for baseline CMR measurements (1 week post MI) and were followed-up at 1 and 3 weeks thereafter (2 and 4 weeks post MI, respectively) and then sacrificed.

### ASC isolation, characterization and preparation for infusion

Subcutaneous adipose tissue was harvested from the neck of healthy pigs and processed for ASC isolation and expansion under hypoxic conditions [7]. The day before infusion, the expanded ASCs were washed and serum-free medium was added. After 24 h, the secretome of ASCs released to the medium (CM) was collected, centrifuged and filtered. Then 30 ml of sample was kept at 4 °C until infusion. ASCs (1 × 10^7^ cells) were resuspended in 2 ml PBS and kept at 4 °C until intracoronary administration. Previously, ASCs had been characterized by cell surface marker expression by flow cytometry and differentiation potential to mesenchymal cell lineages. Aliquots of ASCs and CM were kept for proteomics.

### In-vitro ASC function: proof-of-principle characterization

- 3D cultures: ASC endothelial cell differentiation was evaluated by capillary network formation with HMEC-1 (microvascular endothelial cells) on coculture in 3D matrigel plugs.
- Chorioallantoic membrane assay (CAM) immunofluorescence: fertilized chicken eggs were incubated for 3 days. A small opening was then made in the shell, exposing the CAM. The window was covered with cellophane tape and the eggs were returned to the incubator. Six days later, growth factor-reduced matrigel droplets (containing 10^5^ ASCs, 20 μl concentrated CM (×10) or 10^5^ ASCs + 20 μl concentrated CM) were applied onto the CAM while placement onto big pre-existing blood vessels was avoided. Matrigel droplets containing 20 μl PBS served as negative controls. Following 2 days of incubation the eggs were opened and photographed, and the CAM was carefully dissected and processed for histological analysis against von Willebrand factor (vWF).
- Microvesicle release: ASC-GFP+ microvesicles release into the CM were analyzed by flow cytometry.

### Experimental model of MI

MI was induced by 90-min total balloon occlusion of the LAD as described previously [25]. All animals underwent transthoracic echocardiography before inducing ischemia and upon reperfusion to monitor the impact of MI induction on LVEF [26].

### GFP-lentiviral transduction of ASCs: assessment of ASC cardiac homing

We performed a substudy (*n* = 4 pigs) to confirm ASC retention and homing within the infarcted region post infusion. To that end, ASCs were transduced with GFP-expressing lentiviral vectors and 1 × 10^7^ASC-GFP+ cells were intracoronary infused into MI-induced pigs. Animals were sacrificed 24 h later and samples from multiple cardiac regions (left and right ventricle, ischemic and remote myocardium, atrium) and vascular beds (coronary arteries and different aortic regions) were rapidly obtained for GFP detection.

### Cardiac magnetic resonance imaging

CMR analysis was performed serially as reported previously [25] 1 week post MI just after ASC, CM, ASC + CM or PBS infusion (baseline), 1 week post infusion (2 weeks post MI) and 3 weeks post infusion (4 weeks post MI). The following CMR sequences were acquired: “cine” (b-SSFP) imaging sequence to assess wall motion and cardiac function; and late gadolinium enhancement (LGE) to assess the amount and extent of myocardial necrosis. CMR images were analyzed using dedicated software by a CMR-trained cardiologist blinded to the study medication [27].

### Assessment of myocardial vascular density and angiogenesis

Neovessel formation was assessed by lectin staining and via the analysis of angiogenic markers including gene levels of CD105, vWF, endothelial nitric oxide synthase (eNOS), vascular endothelial growth factor receptor type 2 (VEGFR2), VEGF1, CD31, CD62 and tissue factor (TF) as well as protein activation and/or expression of eNOS phosphorylated in Thr^495^, eNOS, CD105 and vWF.

### Myocardial fibrosis

Myocardial fibrosis was assessed at a transcriptional level (analysis of transforming growth factor beta receptor (TGFβR), TGFβ, and collagen type I and type III) and by histological Masson’s trichromic staining.

### Proteomic analysis

Proteomic analysis was performed on ASCs and their secretome (CM). For all proteomic analyses, protein extracts were separated by bidimensional gel electrophoresis (2-DE) and protein spots of interest were identified by matrix-assisted laser desorption/ionization–time of flight (MALDI-TOF/TOF) as described previously [28].

### In-silico bioinformatics analysis

The statistically significant functional networks in which the identified proteins were involved were generated through the use of ingenuity pathway analysis (IPA; Ingenuity Systems, www.ingenuity.com). The functional analysis of a network identified the biological function and/or disease that were most significant to the molecules in the network. The network molecules associated with biological functions and/or diseases in the Ingenuity Knowledge Base were considered for the analysis.

### Statistical analysis

The Shapiro–Wilk test was applied to verify the normal distribution of the data and statistical analysis was applied accordingly. Within the porcine studies, data were analyzed by a nonparametric statistical analysis and results are reported as medians and interquartile range (IQR). For independent factors (comparisons between groups) we performed Mann–Whitney analysis; for repeated measurements, Wilcoxon and Friedman analyses were appropriate. For the chicken egg analysis we applied a one-way ANOVA followed by Bonferroni’s multiple comparison test. Finally, for in-silico bioinformatics analysis, a right‐tailed Fisher’s exact test was used to calculate a *p* value determining the probability that each biological function and/or disease assigned to that network is due to chance alone.

All statistical tests conducted were two-sided and *p* < 0.05 was considered significant. Statistical analyses were performed with Statview.

## Results

### ASC characterization

**Fig. 1:**
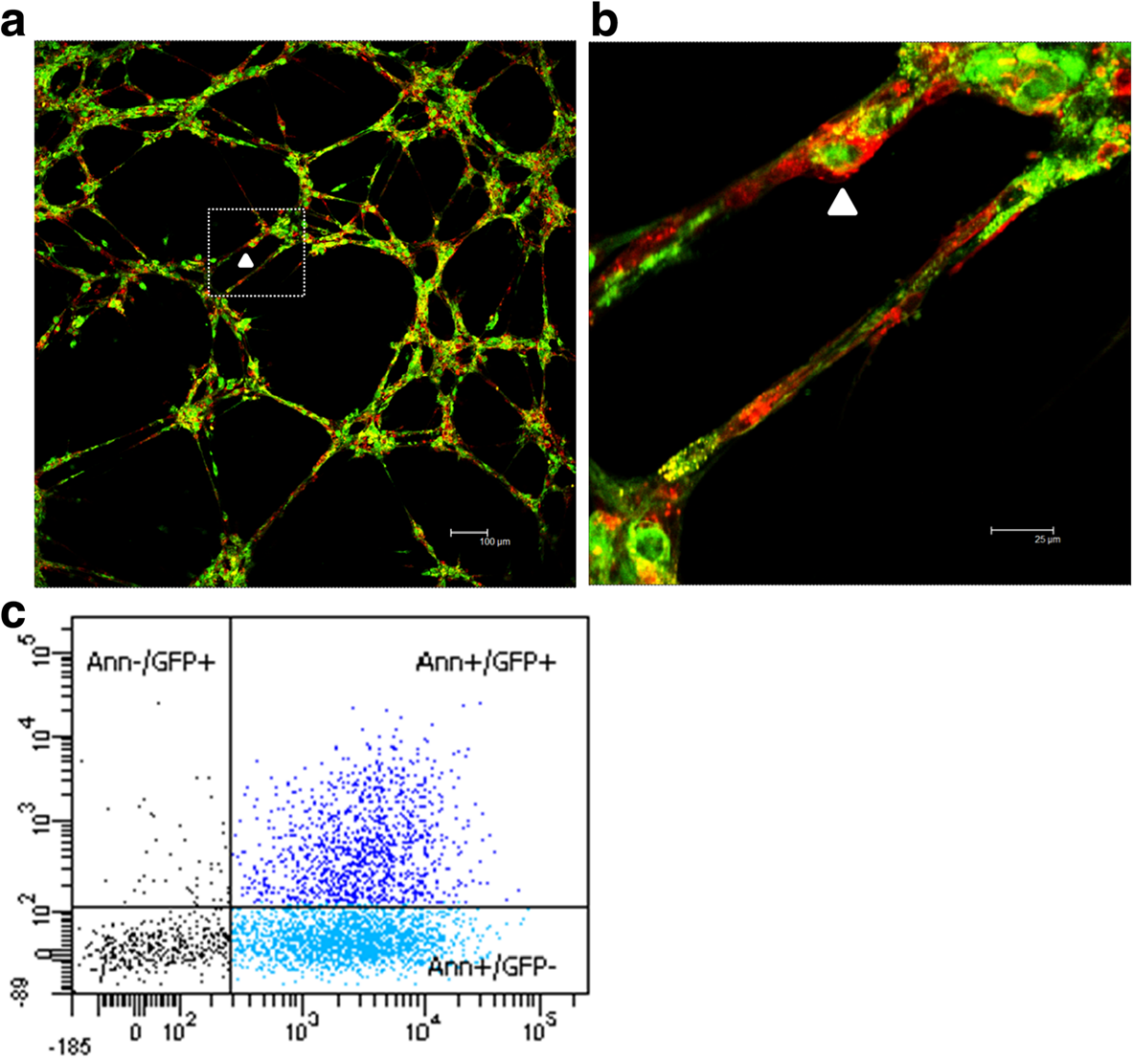
ASC characterization. **a**. ASCs (*green*) and endothelial cells (*red*) in a 3D basement membrane coculture system for 24 h. **b**. Magnification to visualize ASC incorporation (*white arrowhead*) within the capillary network. **c**. Flow cytometry analysis of microvesicles contained in the CM. *Ann* Annexin V, *GFP* green fluorescent protein

Flow cytometry demonstrated that cultured swine ASCs were positive for surface markers characteristic of ASCs including CD105 (99 ± 1%), CD29 (78 ± 4%) and CD90 (90 ± 3%) and negative for CD45 (1.2 ± 0.1%), thereby expressing the mesenchymal cell specific markers reported by our group and others [6, 29]. ASC differentiation potential to mesenchymal cell lineages was assessed. As shown in Additional file 2, after 21 days of induction with specific differentiation medium, staining for lipids and calcium deposition confirmed the differentiation of ASCs towards adipogenic and osteogenic lineages, respectively. Finally, we performed a 3D coculture system of ASCs with endothelial cells to further demonstrate the capacity of ASCs to migrate, contact and localize around the endothelial cells, providing support and stability to the developed capillary-like structures (Fig. 1a, b). Analysis of CM evidenced the presence of ASC-released microvesicles, of which 30% were positive for both Annexin V and GFP (Fig. 1c).

### Chick CAM model of angiogenesis

**Fig. 2:**
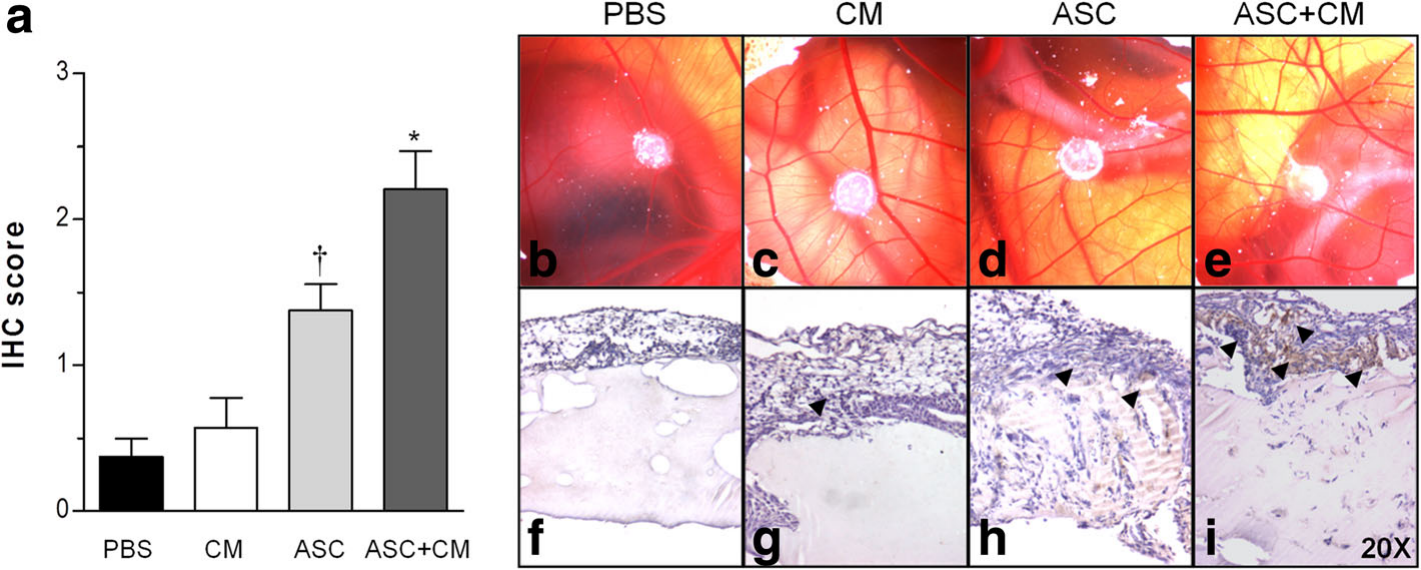
Chick CAM model of angiogenesis. **a** Immunohistochemical (*IHC*) score for vWF staining. Each sample was analyzed double blind and independently by two investigators. Staining (*brown*) was semiquantitatively scored as 0, 1, 2 or 3 for absence, weak, moderate and strong staining, respectively. **b**–**e** Representative macroscopic pictures of 48-h incubated matrigel scaffolds in the in-vivo CAM assay. **f**–**i** Representative immunochemical staining of vWF in sections of matrigel–CAM junction. *p < 0.05 vs PBS, CM and ASCs; †*p* < 0.05 vs PBS. *ASC* adipose-derived mesenchymal stem cell, *CM* conditioned media, *PBS* phosphate-buffered saline

As shown in Fig. 2, ASC + CM seeding in the scaffolds induced a 2-fold and 4-fold increase in angiogenesis in the border area between the CAM and the scaffolds as compared with the ASCs or CM alone as detected by vWF staining (*p* < 0.05). ASCs alone also induced an angiogenic response that reached significance versus control (*p* < 0.05) but was not as great as combined treatment. CM seeding did not induce CAM angiogenesis.

### ASC homing

As observed in Additional file 3A, GFP was detected by RT-PCR in the LAD and in the layers of the ischemic damaged myocardium (from endocardium to epicardium) at 24 h post delivery of ASCs. In contrast, it was undetectable in the remote myocardium, circumflex and right coronary artery, atria, right ventricle and the aorta. Confocal microscopy analysis revealed the presence of GFP+ cells in the LAD and in the ischemic cardiac tissue (Additional file 3B). GFP+ cells were not detected in the nonischemic myocardium (image not shown).

### Cardiac function and damage

Echocardiography revealed that all animals showed a comparable impairment in the left ventricle ejection fraction (LVEF; average 29 (27–30)% decrease) after 90 min of complete coronary ischemia (*p* < 0.05 vs prior MI). Similarly, no variations were detected in hemodynamic parameters (heart rate and mean blood pressure) during MI induction among all groups (average: 76 (74–76) bpm and 56 (54–56) mmHg, respectively).

**Fig. 3:**
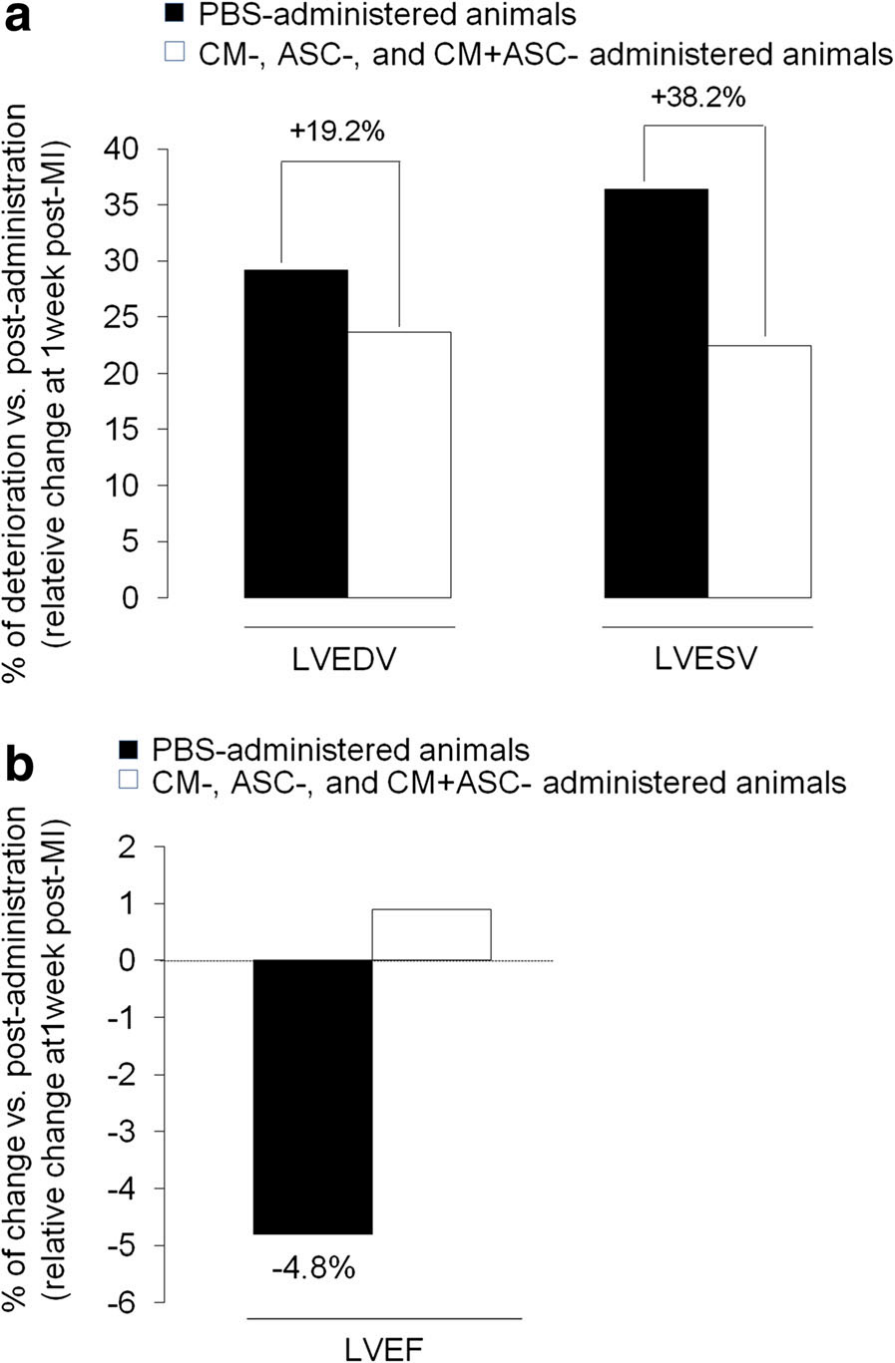
Cardiac function analysis. Changes in cardiac volumes (**a**) and contractility (**b**) 3 weeks post PBS, CM, ASC or CM + ASC administration expressed as relative percentage change vs post infusion. *ASC* adipose-derived stem cell, *CM* ASC conditioned media, *LVEF* left ventricular ejection fraction, *LVEDV* left ventricular end-diastolic volume, *LVESV* left ventricular end-systolic volume, *MI* myocardial infarction

CMR analysis at 1 week post MI (baseline) showed that all groups displayed a comparable deterioration in LVEF and size of infarction. At this time point allogenic cell preparations were administered by coronary infusion of placebo solution or ASCs and intravenous injection of placebo or CM (Table 1). Follow-up CMR analysis showed a deterioration in cardiac contractility in control animals 3 weeks post infusion as compared with baseline, with a marked impairment in left ventricular end-diastolic volume (LVEDV; 38.2% worse), left ventricular end-systolic volume (LVESV; 19.2% worse) and LVEF (4.8% worse) vs the other groups (*p* < 0.05; Fig. 3a, b). Such cardiac deterioration was not detected in those animals administered CM, ASCs or ASCs + CM in which cardiac performance remained unchanged as compared with baseline (Table 1). No differences were detected in the size of the scar at 3 weeks post infusion among the different animal groups (Additional file 4A). Histopathological analysis of the scar by triphenyl tetrazolium chloride (TTC) staining showed reparative fibrosis by increase collagen in the scar area which highly correlated with the CMR data (*r* = 0.85; *p* < 0.01; Additional file 4B).

**Table 1:** MRI analysis of cardiac performance and scar size

		Baseline (1 week post MI)	1 week post infusion (2 weeks post MI)	3 weeks post infusion (4 weeks post MI)
LVEF (%)	PBS	48.4 (48.3–50.1)	48.9 (46.6–50.7)	46.5 (46.3–48.1)*
	ASCs	47.7 (47.6–54.2)	48.3 (47.5–54.1)	48.7 (47.1–49.7)
	CM	47.2 (46.5–47.5)	48.2 (48.0–49.4)	49.5 (48.2–52.1)
	ASCs + CM	50.0 (46.4–53.0)	49.5 (48.7–51.0)	47.6 (47.3–49.6)
LVEDV (ml)	PBS	91.9 (85.3–94.1)	107.5 (101.0–109.1)*	118.7 (115.0–125.7)*†
	ASCs	113.0 (105.0–116.0)	123.2 (101.3–124.3)	121.0 (117.3–129.3)
	CM	87.7(81.9–122.8)	93.4 (89.0–94.1)*	119.2 (106.1–122.3)
	ASCs + CM	91.4 (89.0–98.4)	93.7 (84.0–124.4)	136.32 (82.1–153.0)
LVESV (ml)	PBS	44.0 (40.0–46.2)	55.2 (46.1–58.3)	63.4 (45.0–65.2)†
	ASCs	55.0 (49.9–60.7)	64.4 (51.4–64.9)	62.3 (60.6–66.0)
	CM	42.9 (40.9–64.8)	47.2 (46.0–49.4)	60.2 (47.5–61.5)
	ASCs + CM	46.1 (40.4–50.3)	48.0 (42.0–49.5)	64.8 (43.3–77.0)
Scar size (g)	PBS	11.6 (10.5–12.4)	10.8 (10.1–13.2)	10.4 (9.8–11.7)
	ASCs	9.9 (9.0–16.1)	9.5 (9.4–13.9)	10.8 (9.6–14.4)
	CM	11.3 (9.7–13.2)	10.1 (9.2–11.0)	9.5 (8.1–9.9)
	ASCs + CM	13.0 (10.0–15.0)	12.3 (10.2–14.0)	10.8 (7.2–11.5)

*ASC* adipose-derived stem cell, *CM* ASC conditioned media. *LVEF* left ventricular ejection fraction, *LVEDV* left ventricular end-diastolic volume, *LVESV* left ventricular end-systolic volume, *MI* myocardial infarction, *MRI* magnetic resonance imaging, *PBS* phosphate-buffered saline

*p < 0.05 vs baseline

†*p* < 0.05 vs time

### Vessel density

**Fig. 4:**
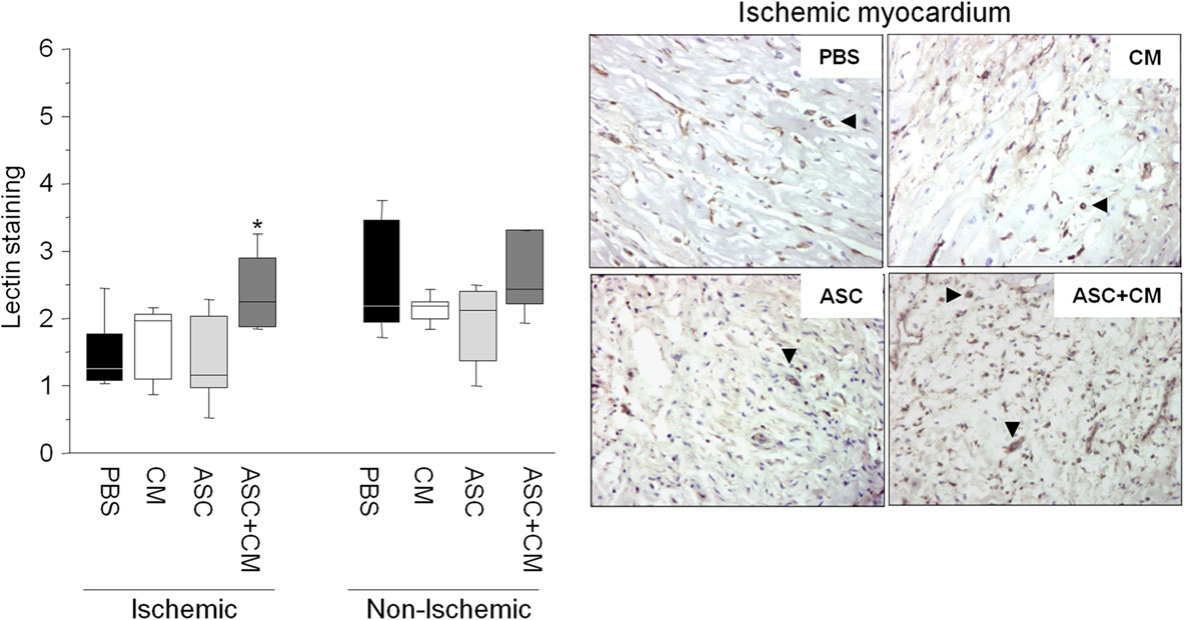
Vessel density analysis of the ischemic and nonischemic myocardium assessed by lectin staining and representative images of the ischemic cardiac tissue. *p < 0.05 vs ASCs, ASC releasate and PBS within the ischemic cardiac tissue. *ASC* adipose-derived stem cell, *CM* ASC conditioned media, *PBS* phosphate-buffered saline

Total vessel density measured by histology operators was found enhanced by 40% in the ischemic myocardium of animals that received the combination of ASCs and CM as compared with ASCs or CM alone or placebo-control (*p* < 0.05; Fig. 4). The amount of vessels detected in the infarcted area of ASC + CM animals was comparable with that observed on the remote nonischemic myocardium.

### Markers of neovessel formation

**Fig. 5:**
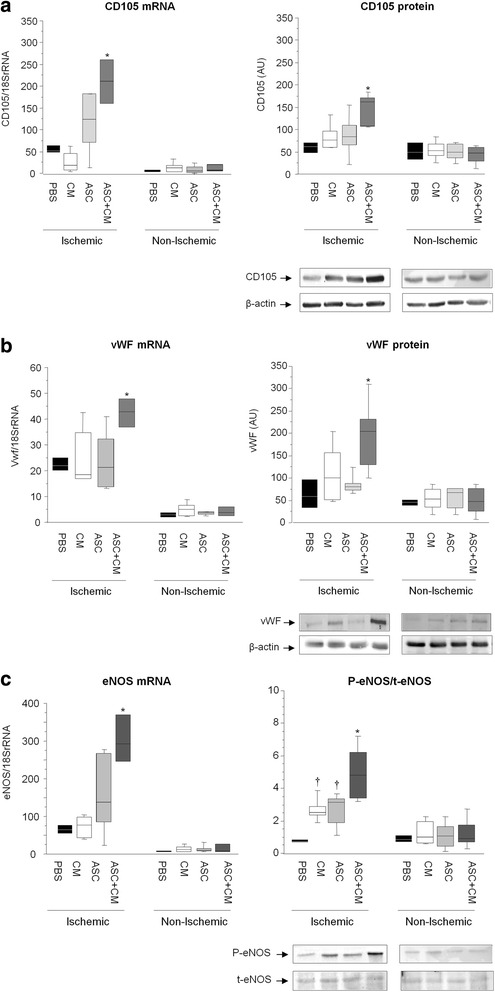
Molecular analysis (gene and protein expression) of markers involved in neovessel formation within the cardiac tissue: **a** CD105, **b** von Willebrand factor (*vWF*) and **c** endothelial nitric oxide synthase (*eNOS*). *p < 0.05 vs ASC, CM and PBS within the ischemic cardiac tissue; †*p* < 0.05 vs PBS within the ischemic myocardium. *ASC* adipose-derived stem cell, *CM* ASC conditioned media, *PBS* phosphate-buffered saline

**Fig. 6:**
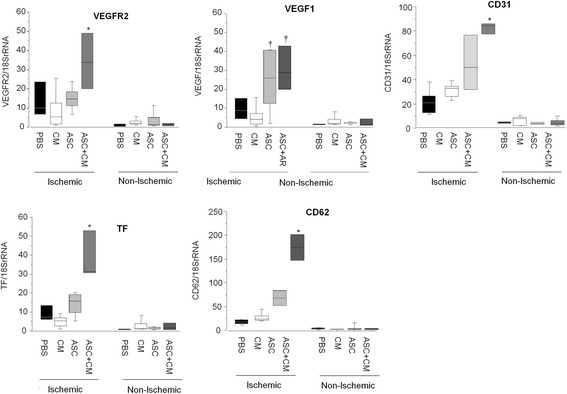
Myocardial gene expression of several markers involved in new vessel formation. *VEGFR2* vascular endothelial growth factor receptor 2, *TF* tissue factor, *CD62* P-selectin, *CD31* platelet. *p < 0.05 vs ASCs, CM and PBS within the ischemic cardiac tissue; †*p* < 0.05 vs CM and PBS within the ischemic cardiac tissue. *ASC* adipose-derived stem cell, *CM* ASC conditioned media, *PBS* phosphate-buffered saline

We next determined myocardial gene and protein expression of markers involved in new vessel formation. CD105 (Fig. 5a) and vWF (Fig. 5b) gene expression and protein levels were significantly higher in the ischemic myocardium of those animals administered ASCs + CM as compared with ASCs or CM alone and PBS control (*p* < 0.05). Moreover, not only eNOS transcription levels but also eNOS activity was markedly enhanced within the ischemic myocardium of animals administered ASCs + CM (*p* < 0.05 vs the other three groups; Fig. 5c). Consistent with these observations, transcription of VEGFR2, TF, CD62 and CD31 was also found to be significantly increased in those animals receiving ASCs + CM (Fig. 6), further supporting, at a molecular level, a proangiogenic synergistic effect of ASC and ASC releasate (CM) administration. VEGF1 was found to be upregulated by ASCs + CM and ASCs (Fig. 6).

### Reparative fibrosis

**Fig. 7:**
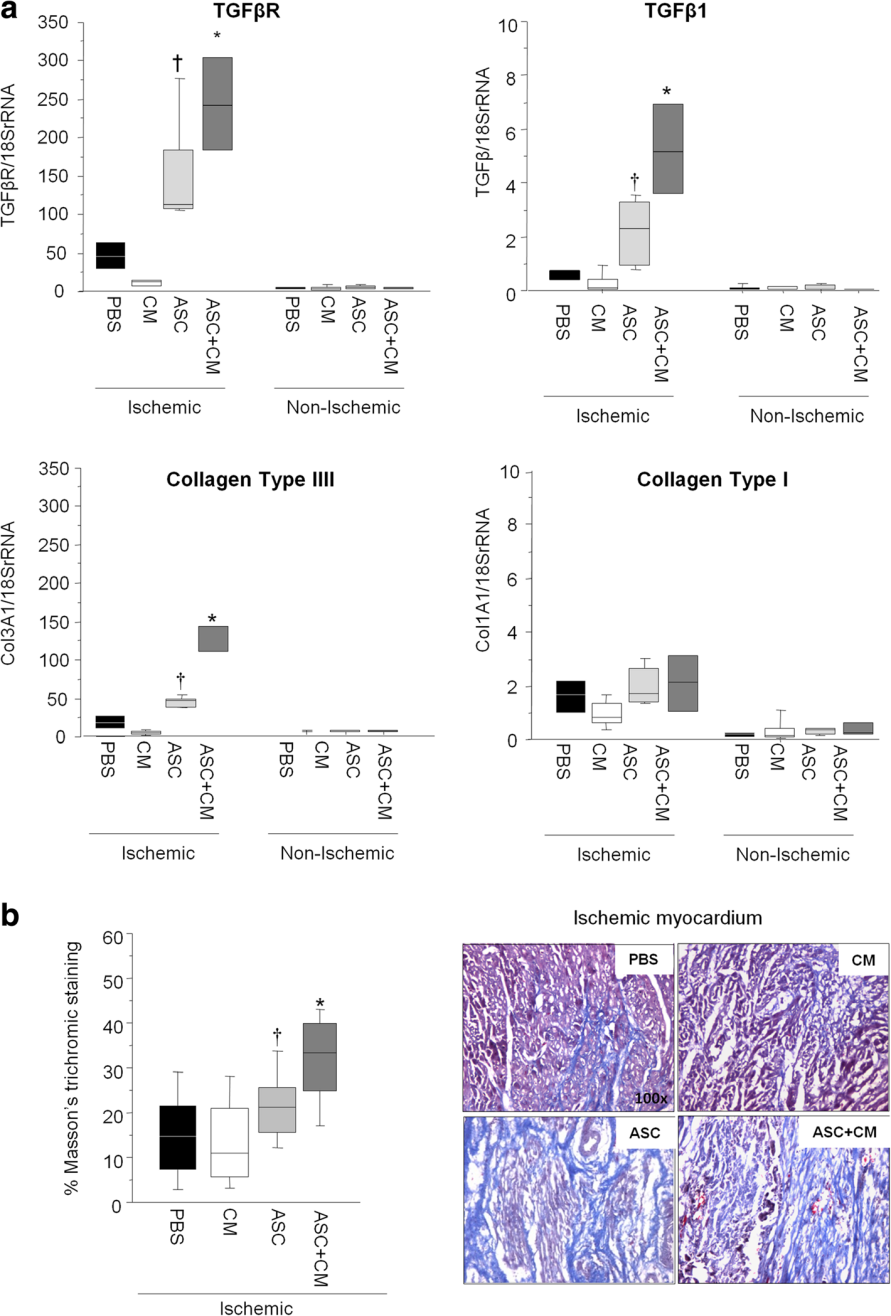
Transcript levels of fibrotic markers (**a**) and collagen deposition in the ischemic myocardium assessed by Masson’s trichromic staining (**b**). *p < 0.05 vs all; †*p* < 0.05 vs CM and PBS within the ischemic cardiac tissue. *ASC* adipose-derived stem cell, *CM* ASC conditioned media, *PBS* phosphate-buffered saline

Gene expression of TGFβR/TGFβ/collagen type III (Fig. **7**a) and collagen deposition (Fig. **7**b) was found to be upregulated in the evolving scar of animals administered ASCs as compared with animals administered PBS and CM (*p* < 0.05). This effect was further enhanced in animals coadministered ASCs + CM (*p* < 0.05 vs ASCs alone). Fibrosis was barely detectable in the nonischemic myocardium (Fig. 7a).

### ASC-related proteome

In order to elucidate the potential mediators by which coadministration of ASCs and CM enhance neovessel formation we analyzed the ASC-related proteome, which included the ASC secretome (CM) and the ASC proteome (ASC cytosol and membrane fractions).

**Fig. 8:**
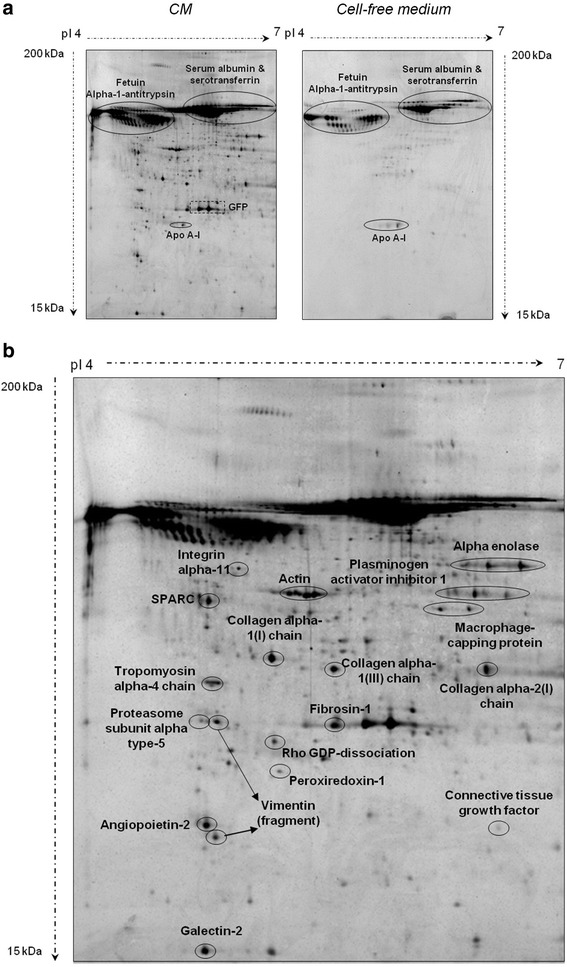
Comparative proteomic analysis between CM and cell-free culture medium. *CM* conditioned media **a**. ﻿Fetuin alph﻿a-1 antitrypsin, ApoA-I, and serum albumin and serotransferrin identification. **b**.﻿ Proteins released by ASCs into the media during cell culture [ASC secretome or conditioned media (CM)]

- ASC secretome analysis: we performed a comparative proteomic approach between CM and cell-free culture medium (negative control). Fetuin alpha-1 antitrypsin, ApoA-I, serum albumin and serotransferrin were identified in both CM and cell-free culture medium and therefore their presence was attributed to the culture medium (Fig. 8a). As expected, GFP was solely identified in the secretome of ASCs and its detection supported its extracellular release.

Figure 8b depicts the proteins released by ASCs into the media during cell culture (ASC secretome). As shown in Additional file 5, we identified proteins related to five functional categories with potential paracrine properties within the CM. These functional groups were related to angiogenesis, cell proliferation/differentiation/apoptosis regulation, protein processing/chaperone activity and structural proteins.

**Fig. 9:**
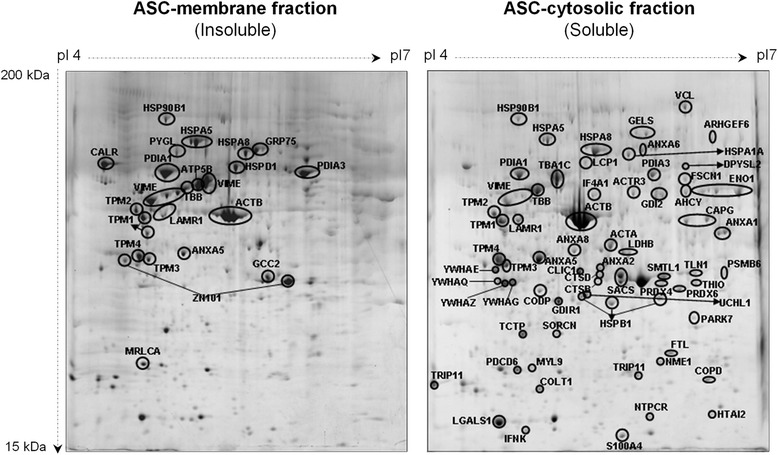
Proteomic analysis of ASC membrane fraction and ASC cytosolic fraction. *ASC* adipose-derived stem cell
c) ASC interactome: we analyzed the functional groups of proteins identified in both fractions (CM and ASCs; Additional file 5) in order to determine the potential interactions (i.e., interactome) behind the synergistic effect observed upon ASC + CM administration. Table 2 presents the functional groups of the proteins identified in the ASC-related proteome. We observe that structural proteins are found in both the ASC secretome and the ASC proteome, although with a differential protein contribution of each fraction. Additionally, the ASC secretome mainly contained proteins related to angiogenesis and cell proliferation/differentiation/apoptosis regulation whereas the ASC proteome was mostly represented by proteins involved in protein processing regulation and chaperone activity.

b) ASC proteome analysis: we further analyzed ASC cytosolic and membrane fractions (Fig. 9). Proteins identified in the ASC proteome (Additional file 5) were related to 11 different functional categories: angiogenesis, antioxidant/redox homeostasis, cell proliferation/differentiation/apoptosis regulation, coagulation/hemostasis, defense response, metabolism, protein processing/chaperone activity, proteolysis, signaling/gene transcription, structural and transport/trafficking proteins.

**Table 2:** Functional groups in the ASC interactome and the percentage of identified proteins

Functional group in donor cell	CM (%)	ASC (%)
Angiogenesis	17	5
Antioxidant/redox homeostasis	0	4
Cell proliferation/differentiation/apoptosis regulation	28	12
Coagulation/hemostasis	0	1
Defense response	0	1
Metabolism	0	3
Protein processing/chaperone	6	22
Proteolysis	0	3
Signaling/gene transcription	0	14
Structural	50	26
Transport/trafficking	0	8

*ASC* adipose-derived stem cell, *CM* conditioned media

We further analyzed the functional networks in which the proteins identified in the ASC interactome were involved by performing an in-silico bioinformatic analysis using the IPA software. This analysis revealed that the most representative network in the ASC interactome was related to blood vessel development and neovascularization (Additional file 6).

## Discussion

Common risk factors associated with coronary artery disease have been shown to attenuate the functional activity of progenitor/adult stem cells likely restricting the effectiveness of autologous cell-based therapy. Therefore, delivery of allogenic stem cells with very low immunogenic reactivity may become a therapeutic option to enhance stem cell potential. Among all kind of stem cells, MSCs and, particularly, ASCs exert considerable immunomodulatory properties and exhibit a low immunogenic profile [16]. ASCs have so far demonstrated encouraging data in several preclinical studies and have proven safety in phase I and phase IIb clinical trials [30, 31]. Autologous ASC therapeutic potential is mainly explained by the production of bioactive molecules that mediate neovascularization, cell survival and proliferation [32]. Yet little is known about the mediators involved and their mechanisms of action. Moreover, key questions remain on the best cell-based preparation, delivery method, dosage and timing. In this study we demonstrate, by immunohistochemical and molecular approaches, that both ASCs and their secretome have beneficial effects. Intravenous administration of CM with an intracoronary infusion of ASCs enhances neovessel formation in the ischemic myocardium as compared with the delivery of CM or ASCs alone. We also identify, by proteomic approaches, the protein networks that may potentially contribute to the detected proangiogenic synergistic effect. Finally, we provide evidence of a positive synergistic effect between ASCs and CM over the evolving reparative scar.

Despite relative enthusiasm arising from rodent data, application of MSC therapy has shown limited clinical benefits in the setting of MI. Concerning ASCs, some preclinical studies have reported a slight amelioration in cardiac contractility [32, 33] whereas others have shown no changes in LVEF at 4 weeks post administration [16, 34]. Recent data from the APOLLO and the PRECISE trials (phase I/IIb trials) suggest that administration of ASCs preserves cardiac function [30, 31]. In our study LVEF is preserved in those animals that received intravenous CM and/or intracoronary ASCs 3 weeks post infusion in contrast to placebo-control animals whose cardiac contractility is found to be significantly deteriorated. Moreover, end-systolic and end-diastolic volumes are worse in PBS-control animals as compared with animals administered ASCs or CM, suggesting ASC-related benefits in cardiac contractility and remodeling. Interestingly, results from the present study reveal that a single intravenous delivery of CM obtained from ASCs (1 × 10^7^ cells) exerts similar protective effects to that of ASCs in cardiac performance (CMR detected parameters) but not in the recovery of the rarefaction (loss of microvessels) where the effect is improved by ASCs plus secretome administration. Administration of stem cells capable of directly inducing vessel formation or secreting proangiogenic factors holds great promise to repair the ischemic damaged tissue. The ability of ASCs to promote angiogenesis has been assessed in multiple preclinical MI experimental animal models [16, 17, 19]. In fact, evidence indicates that ASCs have higher angiogenic capacities than bone marrow MSCs [35, 36]. Several reports have suggested that ASCs increase vascularity by differentiation into pericytes (cells capable of stabilizing the microvasculature) both in vitro and in vitro [37, 38] but ASC transdifferentiation into the endothelial cell lineage has not been shown [22, 23, 34], attributing their angiogenic potential to a paracrine activity [17]. We demonstrate through two different in-vivo models that a combination of CM plus ASCs significantly enhances neoangiogenesis as compared with CM or ASCs alone, suggesting a synergistic interaction among ASCs and CM. Indeed, we detect in the ischemic myocardium a significant increase in both microvessel density and the expression of different angiogenic markers. As such, mRNA transcripts and protein levels reveal that, as compared with animals administered ASCs, CM or PBS, pigs treated with ASCs + CM express: a higher amount of VEGFR2 (the receptor of one of the most potent inducers of angiogenesis, VEGF); specific inducers and markers of active neovessel formation (TF and CD105, respectively) [39]; and a higher number of endothelial cells (P-eNOS, vWF, CD31, CD62) in the ischemic cardiac tissue. Interestingly, however, our results also highlight the ability of ASCs alone to enhance VEGF expression, an effect not observed in CM-alone administered animals. These data indicate a differential contribution of ASCs and CM to neovessel formation, as further discussed in the following.

The increased vascular density and molecular expression of active neoangiogenic/vascular factors detected when combining ASCs and CM at 3 weeks may translate into the amelioration of cardiac contractility in a longer-follow-up period, as seen at 3 months post infusion with autologous cells [19]. Of note, the limited size of infarction with the concomitant mild reduction in ejection fraction may have underestimated the acute potential benefits afforded by ASC + CM administration. Further studies are required to determine the effects of ASCs + CM on LVEF and cardiac remodeling upon a more severe ischemic insult and after longer survival periods.

No consensus exists as per the optimal timing of stem cell administration. We choose a 7-day delay between acute MI and ASC/CM administration based on the following considerations: to closely match clinical trial protocols [40]; to limit interactions with the “cardiac niche effect” favored by MI (progenitor cell recruitment); and to avoid further acute cardiac damage due to ASC-related coronary plugging [41]. In this respect, intracoronary administration of 1 × 10^7^ ADSCs in pigs has been shown to induce the no-reflow phenomenon [32] as well as to compromise coronary microcirculation [42]. These observations support the coadministration, by peripheral delivery, of soluble secreted molecules to increase the benefit of ASC therapy, avoiding the risks and detrimental effects associated with the intracoronary infusion of very high amounts of ASCs.

A recent study in pigs, however, has reported that neovascularization is found to be enhanced when ASCs are given early after reperfusion as compared with 1 week later, likely through paracrine effects [16]. Based on our findings which demonstrate a significant enhanced neovascularization when combining ASCs plus CM and taking into consideration that ASC preconditioning with low oxygen levels induces a faster growth and enhances ASC and CM angiogenic potential [7], the effect of administering ASCs + CM early after revascularization deserves to be investigated.

The complementary proangiogenic effects detected in the ischemic myocardium after the coadministration of ASCs and CM drove the investigation by proteomic approaches of the potential effector molecules. We have identified the presence of angiogenic and inducers of cell proliferation/differentiation in the ASC secretome. Most interestingly, we have identified different and complementary angiogenic proteins in ASCs and CM, supporting the concept of a combined superior effect of ASCs plus CM. The same occurs for proteins related to cell proliferation and differentiation which are critically involved in neovessel formation. As such, annexin A1, a protein known to mediate VEGF-induced endothelial cell migration by regulating the formation of lamellipodia [43], is only detected in the cytosolic ASC-related fraction whereas peroxiredoxin-1, a protein known to stimulate endothelial cell proliferation and migration by interacting with toll-like receptor 4 (TLR4), is only identified in the ASC secretome [44]. Finally, in contrast to the ASC-related secretome, ASCs provide a high number of proteins implicated in the regulation of protein translation and folding, and with molecular chaperone activity such as heat shock protein (HSP)70-1A and protein disulfide isomerase (PDI) known to be involved in-vivo angiogenesis [45, 46]. Overall, our observations suggest the existence of a synergistic and coordinated protein network in the ASC interactome involved in angiogenesis modulation. Further confirming this hypothesis, in*-*silico bioinformatic analysis of the ASC interactome clearly shows a key role for ASC-related proteins in angiogenesis (Additional file 6).

Besides angiogenesis, ASCs have been shown to exert anti-inflammatory and anti-apoptotic properties eventually favoring the cardiac healing process [20]. Cardiac repair is ultimately characterized by fibrous tissue deposition at the site of cardiomyocyte loss in order to preserve structural integrity and prevent myocardial rupture (reparative fibrosis), particularly in transmural infarcts such as the ones performed in the present study. This process requires a series of coordinated molecular and cellular events in which the TGFβ/TβRII signaling governs myofibroblast-related collagen deposition [47]. We show that administration of ASCs favors TGFβ/TβRII-related collagen synthesis in the evolving scar, helping to maintain myocardial shape and structure. Interestingly, this effect is found to be enhanced when combining ASCs plus CM, further supporting a synergistic cross-talk between ASCs and their secretome and in line with the proteomic-related functional groups. Indeed, we observe that ASCs secrete multiple structural proteins such as vimentin, tropomyosin and actin which may have contributed to cellular assembly favoring the healing repair process. This was confirmed by in-silico bioinformatic analysis that revealed the implication of several structural proteins together with antiapoptotic and prosurvival effectors in the cellular assembly and organization functional network. Importantly, neither ASCs nor CM induced interstitial reactive fibrosis (i.e., deleterious collagen deposition in nonischemic cardiac tissue leading to adverse cardiac remodeling), in agreement with recent observations in infarcted mice transplanted a construct of multilayered ASC sheets [48].

## Conclusion

Peripheral vein administration of ASC paracrine mediators in combination with local coronary delivery of ASCs synergistically contributes to enhance the neovascularization of the infarcted myocardium through multiple effectors that interact through a complementary and coordinated protein network. Further studies should approach the evaluation of effects at periods longer than 3 weeks. In addition, further studies are needed to ascertain which of the released molecules are endowed with beneficial effects in order to improve delivery of purified paracrine regulators of angiogenesis and neovessel formation.

## Additional files

Additional file 1: Is a Word file presenting supplemental materials and methods. (DOC 84 kb)
Additional file 2: Is a figure showing adipogenic and osteogenic differentiation of ASCs. ASC-related differentiation towards mesodermal lineages was determined by specific staining; Alizarin red staining showed extracellular calcium deposition after osteogenic differentiation whereas adipogenic differentiation was evidenced by lipid droplets stained by Oil red. (TIF 8942 kb)
Additional file 3: Is a table and figure showing analysis of ASC homing. (**A**) GFP+ detection in different regions of the heart and aorta by real-time PCR analysis. We set a cycle threshold (CT) of 28 as the cutoff value. (**B**) ASC-GFP+ cells by confocal analysis in the left anterior descending coronary artery and ischemic left ventricle (*red*: smooth muscle actin; *green*: GFP; *blue*: nuclei). Identification was carried out 24 h after intracoronary infusion of ASC-GFP+ cells to the infarcted myocardium. *LAD* left anterior descending coronary artery, *LV* left ventricle. (TIF 5200 kb)
Additional file 4: Is a figure showing correlation between infarct size assessment by histopathology and by CMR in all animals of the study. *CM* adipose-derived stem cell (ASC) conditioned media. (TIF 2419 kb)
Additional file 5: Is Table S1 presenting identified proteins by MALDI-TOF/TOF in ASCs and CM (ASC releasate). (DOC 171 kb)
Additional file 6: Is a figure showing the ASC interactome–angiogenesis network. (TIF 11036 kb)

## Abbreviations

ASC: Adipose-derived mesenchymal stem cell
CAM: Chorioallantoic membrane assay
CM: Conditioned media
CMR: Cardiac magnetic resonance imaging
eNOS: Endothelial nitric oxide synthase
FBS: Fetal bovine serum
HSP: Heat shock protein
IHD: Ischemic heart disease
IPA: Ingenuity pathway analysis
LGE: Late Gadolinium enhancement
LVEDV: Left ventricular end-diastolic volume
LVEF: Left ventricular ejection fraction
LVESV: Left ventricular end-systolic volume
MI: Myocardial infarction
MSC: Mesenchymal stem cell
PDI: Protein disulfide isomerase
TF: Tissue factor
TGF: Transforming growth factor
TLR4: Toll-like receptor 4
TTC: Triphenyl tetrazolium chloride
VEGF: Vascular endothelial growth factor
vWF: Von Willebrand factor
