# Supplementary material for: Allogenic adipose-derived stem cell therapy overcomes ischemia-induced microvessel rarefaction in the myocardium: systems biology study
Source: Stem Cell Res Ther. 2017 Mar 9;8:52. doi: 10.1186/s13287-017-0509-2 (PMC5345145; doi:10.1186/s13287-017-0509-2)
Supplement: Additional file 1: — Is a Word file presenting supplemental materials and methods. (DOC 84 kb) [file 13287_2017_509_MOESM1_ESM.doc]

**Allogenic adipose-derived stem cell therapy overcomes ischemia-induced microvessel rarefaction in the myocardium: Systems biology study.**

**Running title:** Stem cell therapy and systems biology

***By***

Gemma Vilahur^1,2^, Blanca Oñate^1^, Judit Cubedo^1^, M. Teresa Béjar^1^, Gemma Arderiu^1^,

Esther Peña^1,2^, Laura Casaní^1,2^, Manuel Gutiérrez^3^, Antoni Capdevila^3^, Guillem Pons^4^,

Francesc Carreras^4^, Alberto Hidalgo^3^, Lina Badimon^1,2,5^

^1^Cardiovascular Research Center (CSIC-ICCC) Hospital de la Santa Creu i Sant Pau (HSCSP); ^2^ Ciber CV ^3^Radiology Unit (HSCSP), ^4^Cardiology Unit (HSCSP), and ^5^Cardiovascular Research Chair UAB (Autonomous University of Barcelona)

Correspondence to:

**Prof. Lina Badimon**

Cardiovascular Research Center

c/Sant Antoni Mª Claret 167,

08025 Barcelona, Spain.

*Phone*: +34.935565880

*Fax*: +34.935565559

*E-mail*: [lbadimon@csic-iccc.org](mailto:lbadimon@csic-iccc.org)

# SUPPLEMENTAL MATERIALS & METHODS

## Ethical approval

**The experimental procedures with animals were review and approved by the Institutional Animal Care and Use Committees (CEEA-ICCC) and authorized by the Animal Experimental Committee of the local government (# 5601) in accordance to the Spanish law (RD 53/2013) and European Directive 2010/63/EU.** In addition, we have followed the ARRIVE guidelines and committed to the 3Rs of laboratory animal research and consequently used the minimal number of animals to reach statistical significance. All animals were allowed to acclimate 7 days prior any intervention and housed in individual cages under light-controlled conditions and room temperature.

## Experimental design

Pigs (n=20) were subjected to closed-chest 90min coronary balloon occlusion and further reperfusion (MI). 1week post-MI animals were randomized to receive: I) ASC (1x10^7^); II) **conditioned media** (**CM**; 30mL); III) ASCs and their **CM**; or IV) PBS (control; 30mL). ASC coronary delivery was performed with an over-the-wire catheter and total balloon occlusion for 2min. **CM** was administered intravenously (marginal ear vein). Cell preparations were blindly administered by the surgical operators. Then, animals were brought to the CMR facilities for baseline CMR-measurements (1week post-MI) and followed-up at 1week and 3weeks thereafter (2weeks and 4weeks post-MI, respectively) and then sacrificed. Computer analysis was also performed blindly by the cardiology partners.

## ASC isolation, characterization and preparation for infusion

Subcutaneous adipose tissue was harvested from the neck of healthy pigs, minced and incubated with type I collagenase solution (1mg/ml; Sigma-Aldrich, St. Louis, MO) for 30min in a 37 ºC pre-warmed orbital shaker. The suspension was then centrifuged at 300g for 10min to obtain the stromal vascular fraction. The pellet was resuspended in growth medium (DMEM supplemented with 10% FBS and 1% Penicillin-Streptomycin) and filtered through a 100µm mesh filter to remove cellular debris. The cells were plated on conventional cell culture flasks and left overnight at 37 ºC and 5% CO_2_. After 24h, non-adherent cells were removed, fresh medium was added and ASC were allowed to expand **up to passage 4-5** under hypoxic conditions (1% O_2_) for further experiments. We have already proven that hypoxic conditions precondition ASC to grow faster than under normoxia.

The day before infusion, the expanded ASC ready for transplantation were washed exhaustively to remove FBS and serum-free medium was added. After 24h, the secretome of ASC released to the medium (**CM**) was collected, centrifuged (300g for 10min) and filtered through a 0.22μm mesh. 30mL were kept at 4 ºC until infusion through the marginal ear vein. ASCs were trypsinized, washed twice with PBS and 1x10^7^ cells were resuspended in 2mL PBS and kept at 4 ºC until intracoronary infusion. Previously, ASC had been characterized by cell surface marker expression by flow cytometry analyses with antibodies against **CD105**, CD29, CD90 and CD45 (eBioscience, San Diego) according to the manufacturer's protocol. **Additionally, differentiation potential towards mesodermal lineages was assessed. ASCs were seeded at a density of 1.5 x 10^4^ cells/cm2 in 6-well plates and allowed to grow to confluence. Subsequently, cells were allowed to differentiate for 21 days in adipogenic medium (containing 0.5 mM 3-isobutyl-1-methylxanthine, 1 µM dexamethasone, 100 µM indomethacin and 10 µg/ml insulin) or osteogenic medium (containing 2 mM β-glycerophosphate disodium, 10-7 M dexamethasone, 50 µg/ml ascorbic acid and 0.15 mM sodium phosphate). All chemicals were obtained from Sigma-Aldrich. Adipogenic and osteogenic differentiation was evaluated by Oil Red O and Alizarin red staining respectively.**  Aliquots of ASCs and **CM** were kept for proteomics.

**In vitro ASC function: proof-of-principle characterization**

1. ***3D-cultures*: ASCs (passage 1) were cultured with endothelial cell differentiation medium (M-199 supplemented with 3% FBS, 10ng/mL basic Fibroblast Growth Factor (BD Biosciences) and 50ng/ml VEGF (Sigma-Aldrich) for one week. Then, 3D cultures were prepared on 3D basement membrane (BD Matrigel™; BD Biosciences). The co-culture system was run with ASCs and HMEC-1 (human microvascular endothelial cell line) (2x10^5^ cells 1:1 ratio). To discriminate each cell type, cells were labeled with two different living cells fluorescent membrane dyes, PKH67 and PKH26 (Sigma-Aldrich). Cell movement was monitored by time lapse video microscopy at 15-minute intervals. Cells were viewed using a PL APO 20x/0.7 Multiimmersion CS. Images were acquired, digitalized, and processed with Leica Software**
2. *CMA-immunofluorescence:* For the chorioallantoic membrane assay (CMA; see below), AR was concentrated 10x by centrifugation in Agilent Spin concentrator tubes with 5.000 Molecular Weight cut-off (Agilent Technologies, UK).
3. ***Microvesicle*** *release:* We also assessed the release of ASC-GFP+ microvesicles into the **CM**. **CM** was centrifuged at 150.000g for 90min at 4 ºC and the pellet was resuspended in DMEM and further analyzed for Annexin V and GFP markers by flow cytometry.

## Experimental model of MI

MI was induced by closed-chest 90min total balloon occlusion of the mid-left anterior descending (LAD) coronary artery as we have previously described. The day before the experimental procedure animals were administered a loading dose of clopidogrel to avoid catheter-related thrombotic complications. The day after pigs were sedated with an intramuscular injection of tiletamine+zolazepam (7mg/kg)+medetomidine (0.07mg/kg), endotracheally intubated and anesthesia was maintained with isofluorane (2%) during all the experimental procedure. After 90min of ischemia the balloon was deflated and removed allowing blood reperfusion and animals were taken to the recovery room and kept under close surveillance. Animals had continuous electrocardiogram (ECG) and hemodynamic monitoring throughout all procedure. All animals underwent transthoracic echocardiography (Phillips iE33 equipped with a S5-1 Sector Array transducer) before inducing ischemia and upon reperfusion to monitor the impact of MI-induction on LVEF.

## GFP-lentiviral transduction of ASC: assessment of ASC-cardiac homing

We performed a substudy (n=4 pigs) to confirm ASC retention and homing within the infarcted region post-infusion. To that end, ASC were transduced with GFP-expressing lentiviral vectors and 1x10^7^ASC-GFP+ cells were intracoronary infused in MI-induced pigs as reported above. Animals were sacrificed 24h later and samples from multiple cardiac regions (left and right ventricle, ischemic and remote myocardium, atrium) and vascular beds (coronary arteries and different aortic regions) were rapidly obtained and immersed in liquid N_2_ or embedded in OCT for GFP detection by real time-PCR or confocal analysis, respectively.

In order to label the ASC, pLenti6.3/V5-GW/EmGFP Invitrogen (Life Technologies, #V370-06) was transfected in 293FT cells using the lentivirus packaging mix (ViraPowerTM Packaging Mix). 48h after transfection, viral supernatants were collected, passed through 0.45µm filters and used for transduction of ASCs. Cells were infected with pLenti-GFP using 2 mL of virus-containing media in the presence of 10 μg/mL polybrene (Sigma-Aldrich). ASC were incubated for 24h before a second infection was performed following the same protocol. 24h later infected ASCs were selected in 5µg/mL blasticidin, and the pooled cell population was used for subsequent experiments. To evaluate transduction efficiency, ASC-GFP^+^ were analyzed by flow cytometry in a Beckman Coulter Epics XL.

## Cardiac magnetic resonance (CMR)

CMR was performed on a 3.0T-CMR system (Achieva®, Philips) by operators blinded to the study arm as we have previously reported. CMR analysis was performed serially at 1week post-MI just after ASC, **CM**, ASC+ **CM** or PBS infusion (baseline), 1week post-infusion (2weeks post-MI) and at 3weeks post-infusion (4weeks post-MI). Briefly, animals were sedated, maintained by continuous intravenous propofol infusion (2%) and taken to the CMR. Animals were kept under mechanical ventilation during all the procedure. Animals were positioned in a head-first supine position with a flexible phased-array surface coil placed over the chest. ECG gating was used to acquire still images of the heart. The following dedicated CMR sequences were acquired in all cases: “cine” (b-SSFP) imaging sequence to assess wall motion and cardiac function and late gadolinium enhancement (LGE) to assess the amount and extent of myocardial necrosis. All the CMR studies followed the same scheme. First, scout images (T1-TFE sequence) were obtained to localize the true axes of the heart and define a field of view involving the whole heart. Afterward, the b-SSFP cine imaging was performed in both, horizontal and vertical long axes (4-chamber and 2-chamber views) and in multiple contiguous short axis images covering the whole LV. In the short axis cine sequence 24 cardiac phases of every slice were acquired to guarantee a correct evaluation of the wall motion and heart function. Thereafter, a gadolinium-based contrast agent was injected intravenously (Gd-GTPA, Magnevist®) at a dose of 0.1mmol/kg and LGE sequences were obtained ten minutes after the administration of contrast. Once the parameter acquisition was finished animals were heparinised and euthanized.

## CMR data analysis

All CMR images were analyzed using dedicated software (QMass MR v.7.6, Medis) by a CMR-trained cardiologist blinded to the study medication. The analysis protocol has been detailed elsewhere. In brief, LV cardiac borders were traced in each image of the cardiac phases representing the end diastole and end systole to obtain the LVEDV, LVESV and LVEF. LV volumes normalized to the body surface area. Infarct size (necrosis) was quantified from the extent of myocardial enhancement in the LGE-CMR sequence and was identified as a hyperintense region, defined as 50 % of the peak myocardial signal intensity with manual adjustment when needed. In order to establish histopathologic correlation with the 3T-CMR within the next 24h after the last CMR, animal hearts’ were arrested with an intravenous injection of 10mL potassium chloride 2M, rapidly excised and sectioned into 6 transverse slices parallel to the atrio-ventricular ring. Consecutive slices were alternatively collected for histopathological scar size analysis (triphenyltetrazolium chloride staining) and tissue sample collection. Scar size was determined by planimetry by an independent, blinded observer using imageJ® software and was expressed as a %LV.

## Assessment of myocardial vascular density and angiogenesis

At sacrifice cardiac tissue was obtained from ischemic and non-ischemic myocardium and blindly examined for neovessel formation and reparative fibrosis.

Neovessel formation was assessed by lectin staining and via the analysis of angiogenic markers at transcript and protein level. Briefly, myocardial tissues were immersed in 4% paraformaldehyde, embedded in O.C.T._TM_ compound (Tissue-Tek®) and cut into 5mm thick serial sections and placed on poly-L-lysine coated slides. Sections were thawed, the endogenous peroxidase activity was blocked with H_2_O_2_ (in methanol for 30min.) and unspecific BSA blocking was performed. **Lectin was detected using the Biotinylated Griffonia (Bandeiraea) Simplicifolia Lectin I; Isolectin B4 (B-1205; Vector)**. Haematoxylin was used for nuclear staining. Images were captured with a Nikon Eclipse 80i microscope, and digitized by a Retiga 1300i camera. On the other hand, myocardial tissue was homogenized and processed for mRNA and protein isolation. We evaluated multiple markers of neovessel formation at a transcriptional level including CD105, von Willebrand factor (vWF), endothelial nitric oxide synthase (eNOS), vascular endothelial growth factor receptor type 2 (VEGFR2), VEGF1, CD31, CD62, and tissue factor (TF) as well as protein activation and/or expression of eNOS phosphorylated in Thr ^495^, eNOS, CD105, and vWF.

## Myocardial fibrosis

Myocardial fibrosis was assessed at a transcriptional level [analysis of transforming growth factor beta receptor (TGFβR), TGFβ, and collagen type I and type III] and by histological Masson’s trichromic staining. For the latter, collagen deposition was assessed as the percentage of blue staining of 6 consecutive myocardial sections (ischemic and non-ischemic myocardium) per animal (ImageJ, NIH).

## CAM angiogenesis model.

We carried out this *in vivo* experimental approach to further assess ASC and **CM** angiogenic potential. To this end, fertilized chicken eggs (Gallus gallus) were incubated for 3d at 37 °C in a humidified atmosphere. At day 3 of embryonic development (E3), 3–4mL of albumin was removed to detach the egg shell from the developing CAM. A small opening was made in the shell, exposing the CAM. The window was covered with cellophane tape and the eggs were returned to the incubator. Six days later (E9), 30µL growth factor reduced matrigel droplets (Corning, Germany) containing 10^5^ ASCs, 20µl concentrated AR (X10) or 10^5^ ASCs plus 20µL concentrated AR were applied onto the CAM while placement onto big pre-existing blood vessels was avoided. Matrigel droplets containing 20µL PBS served as negative controls. Following 2days of incubation (E11) the eggs were opened and photographed with Optem Zoom 70XL coupled to Qimaging Retiga 1300i Fast1394 camera. Then the CAM was carefully dissected out of the eggs and processed for histological analysis.

For immunohistochemical detection of vessels, 5μm thick slices of paraffin embedded matrigel were incubated for 2h with antibody against vWF (Dako) and detected with avidin–biotin immunoperoxidase technique. Staining was semiquantitatively scored as 0, 1, 2 or 3 for absence-, weak-, and moderate- and strong- staining, respectively, by two independent blinded observers.

## Proteomic analysis

Proteomic analysis was performed on ASCs and their secretome (**CM**). For all proteomic analyses, protein extracts were separated by bidimensional gel electrophoresis (2-DE) and protein spots of interest identified by matrix-assisted laser desorption/ionization-time of flight (MALDI-TOF/TOF) as previously described .

Sample preparation for proteomic analysis: Protein fractions were obtained using the ProteoExtract Subcellular Proteome Extraction Kit (Calbiochem). All protein extracts were desalted using ReadyPrep 2-D Cleanup Kit (BioRad) and resuspended in a urea/thiourea/chaps/DTT buffer. Protein concentration was measured with 2D-Quant Kit (GE Healthcare). Processed samples were stored at -80 ºC until processed.

Two-dimensional gel electrophoresis (2-DE): For analytical and preparative gels, respectively, a protein load of 100µg and 300µg protein of the urea/thiourea/chaps/DTT extracts was applied to 17cm dry strips (pH 4-7 linear range, BioRad). Gels were developed by fluorescent staining (Flamingo stain, BioRad). The AR was determined by the differential analysis of the protein spots detected in ASC-media and in basal media (cell-free media), performed with the PD-Quest software (BioRad). Each spot was assigned a relative value that corresponded to the single spot volume compared to the volume of all spots in the gel, following background extraction and normalization between gels. Spots detected in the **CM** but not in the cell-free media correspond to the secretome of ASC.

Mass spectrometry analysis: Protein spots were blindly excised from 2-DE gels (operators not knowing type of sample), washed, dehydrated, dried, and enzymatic digested with sequence-grade modified porcine trypsin (Promega) and analyzed by matrix-assisted laser desorption/ionization time-of-flight (MALDI-TOF) using an AutoFlex III Smartbeam MALDI-TOF/TOF (Bruker Daltonics). Samples were applied to Prespotted AnchorChip plates (Bruker Daltonics) surrounding the calibrants provided on the plates. Spectra were acquired with flexControl on reflector mode, (mass range 850-4000 m/z, reflector 1: 21.06 kV; reflector 2: 9.77kV; ion source 1 voltage: 19 kV; ion source 2: 16.5kV; detection gain 2.37x) with an average of 3500 added shots at a frequency of 200 Hz. Each sample was processed with flexAnalysis (version 3.0, Bruker Daltonics) considering a signal-to-noise ratio over 3, applying statistical calibration and eliminating background peaks. For identification, peaks between 850 and 1000 were not considered as in general only matrix peaks are visible on this mass range. After processing, spectra were sent to the interface BioTools (version 3.2, Bruker Daltonics) and, with no further modifications, a MASCOT search on Swiss-Prot 57.15 database was performed [(Mass Tolerance 50 to 100, up to 2 miss cleavage, Global Modification: Carbamidomethyl (C), Variable Modification: Oxidation (M)]. Identification by peptide mass fingerprint was confirmed by MS/MS.

## *In-silico* bioinformatics analysis

The statistically significant functional networks in which the identified proteins were involved were generated through the use of ingenuity pathway analysis (IPA; Ingenuity Systems, www.ingenuity.com). The Functional Analysis of a network identified the biological function and/or disease that were most significant to the molecules in the network. The network molecules associated with biological functions and/or diseases in the Ingenuity Knowledge Base were considered for the analysis.

## Statistical analysis

Shapiro-Wilk test was applied to verify the normal distribution of the data and statistical analysis were accordingly applied. Within the porcine studies data was analyzed by a non-parametric statistical analysis and results are reported as medians and interquartil range [IQR]. For independent factors (comparisons between groups) we performed Mann-Whitney analysis; for repeated measurements Wilcoxon and Friedman analysis were appropriate. For the chicken egg analysis we applied a One-way ANOVA followed by a Bonferroni’s Multiple comparison test. Finally, for *in-silico* bioinformatics analysis, right‐tailed Fisher’s exact test was used to calculate a *P*‐value determining the probability that each biological function and/or disease assigned to that network is due to chance alone.

All statistical tests conducted were two-sided and p<0.05 was considered significant. Statistical analyses were performed with Statview.

***References***

1. Kilkenny C, Browne WJ, Cuthi I, Emerson M, Altman DG. Improving bioscience research reporting: the ARRIVE guidelines for reporting animal research. PLoS Biol 2010; 8:e1000412.

2. Onate B, Vilahur G, Ferrer-Lorente R et al. The subcutaneous adipose tissue reservoir of functionally active stem cells is reduced in obese patients. FASEB J 2012; 26:4327-36.

3. Vilahur G, Gutierrez M, Casani L et al. Hypercholesterolemia Abolishes High-Density Lipoprotein-Related Cardioprotective Effects in the Setting of Myocardial Infarction. J Am Coll Cardiol 2015; 66:2469-70.

4. Thomas WP, Gaber CE, Jacobs GJ et al. Recommendations for standards in transthoracic two-dimensional echocardiography in the dog and cat. Echocardiography Committee of the Specialty of Cardiology, American College of Veterinary Internal Medicine. J Vet Intern Med 1993; 7:247-52.

5. Ibanez B, Prat-Gonzalez S, Speidl WS et al. Early metoprolol administration before coronary reperfusion results in increased myocardial salvage: analysis of ischemic myocardium at risk using cardiac magnetic resonance. Circulation 2007; 115:2909-16.

6. Vilahur G, Cubedo J, Casani L et al. Reperfusion-triggered stress protein response in the myocardium is blocked by post-conditioning. Systems biology pathway analysis highlights the key role of the canonical aryl-hydrocarbon receptor pathway. Eur Heart J 2012; 34:2082-93.
