# Supplementary figures and images for: Allogenic adipose-derived stem cell therapy overcomes ischemia-induced microvessel rarefaction in the myocardium: systems biology study
Source: Stem Cell Res Ther. 2017 Mar 9;8:52. doi: 10.1186/s13287-017-0509-2 (PMC5345145; doi:10.1186/s13287-017-0509-2)

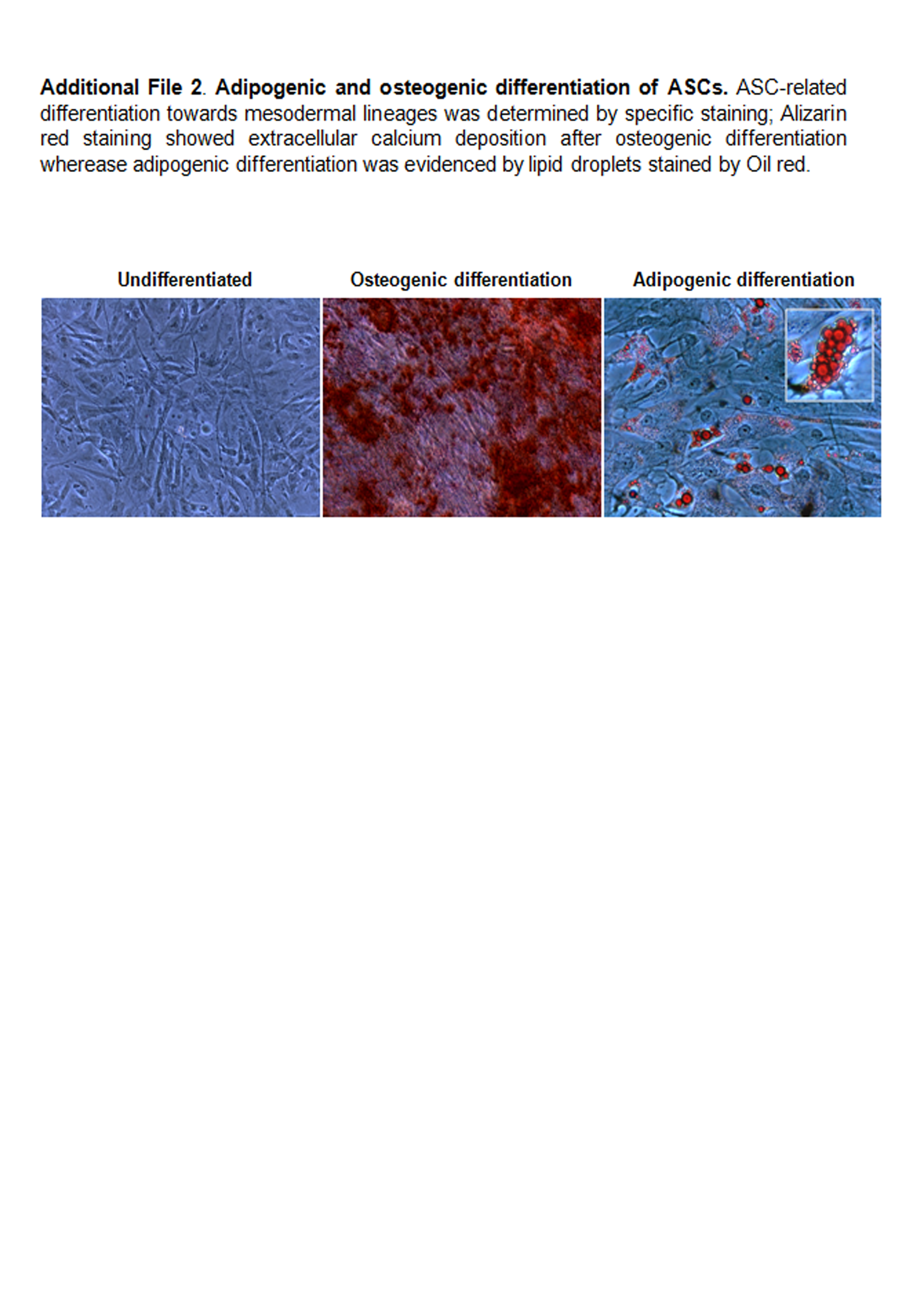

Supplement: Additional file 2: — Is a figure showing adipogenic and osteogenic differentiation of ASCs. ASC-related differentiation towards mesodermal lineages was determined by specific staining; Alizarin red staining showed extracellular calcium deposition after osteogenic differentiation whereas adipogenic differentiation was evidenced by lipid droplets stained by Oil red. (TIF 8942 kb) [file 13287_2017_509_MOESM2_ESM.tif]

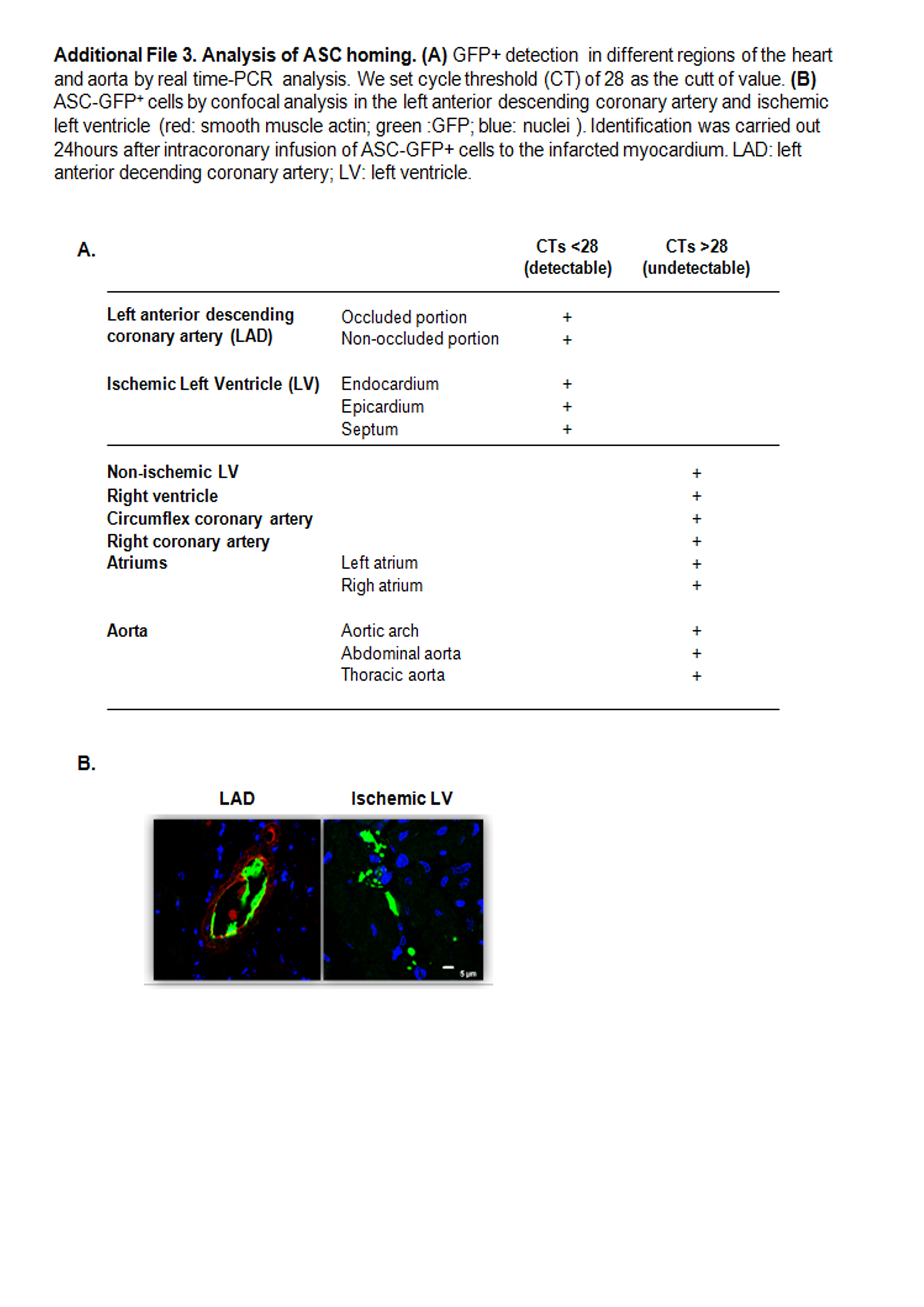

Supplement: Additional file 3: — Is a table and figure showing analysis of ASC homing. (A) GFP+ detection in different regions of the heart and aorta by real-time PCR analysis. We set a cycle threshold (CT) of 28 as the cutoff value. (B) ASC-GFP+ cells by confocal analysis in the left anterior descending coronary artery and ischemic left ventricle (red: smooth muscle actin; green: GFP; blue: nuclei). Identification was carried out 24 h after intracoronary infusion of ASC-GFP+ cells to the infarcted myocardium. LAD left anterior descending coronary artery, LV left ventricle. (TIF 5200 kb) [file 13287_2017_509_MOESM3_ESM.tif]

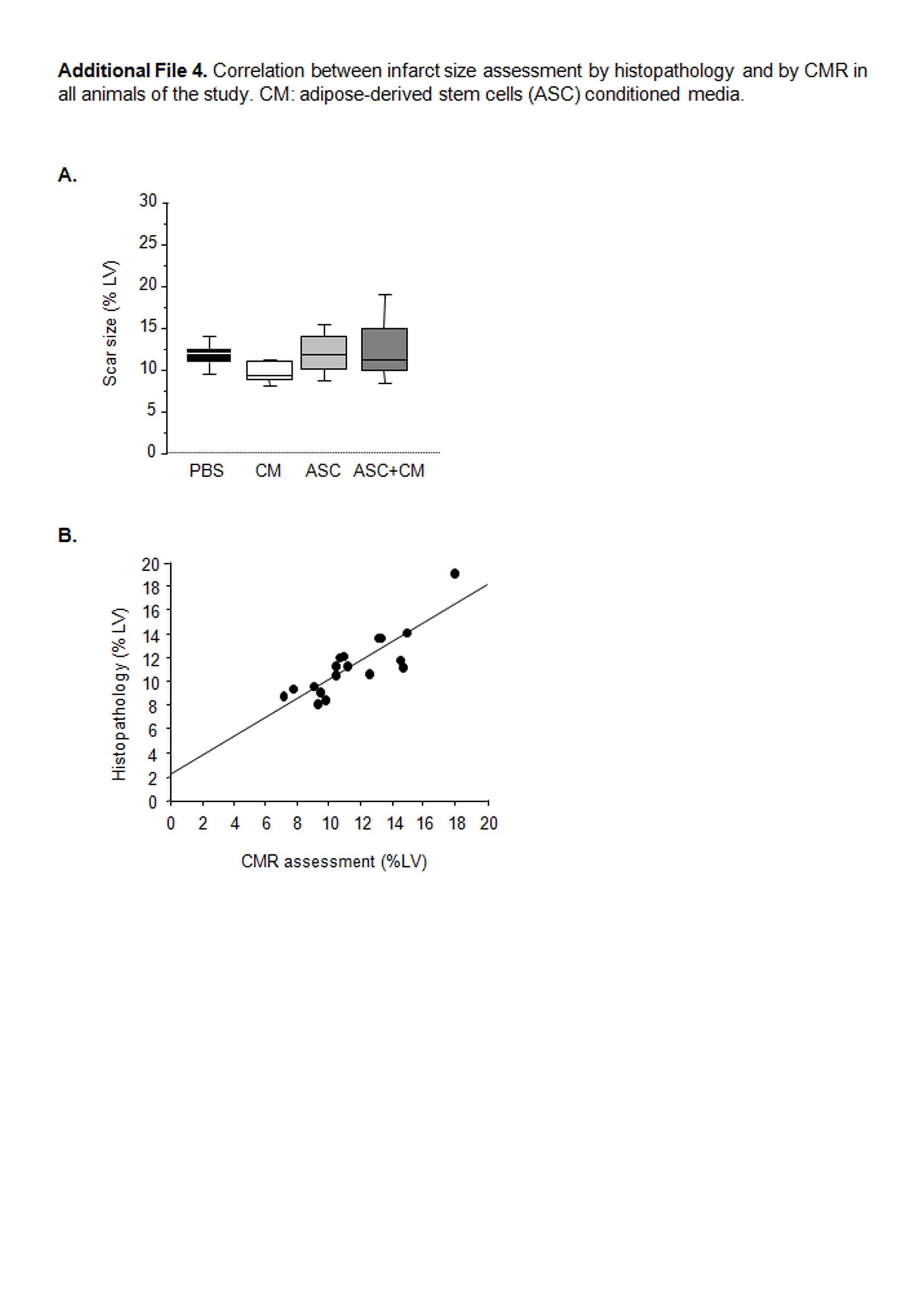

Supplement: Additional file 4: — Is a figure showing correlation between infarct size assessment by histopathology and by CMR in all animals of the study. CM adipose-derived stem cell (ASC) conditioned media. (TIF 2419 kb) [file 13287_2017_509_MOESM4_ESM.tif]

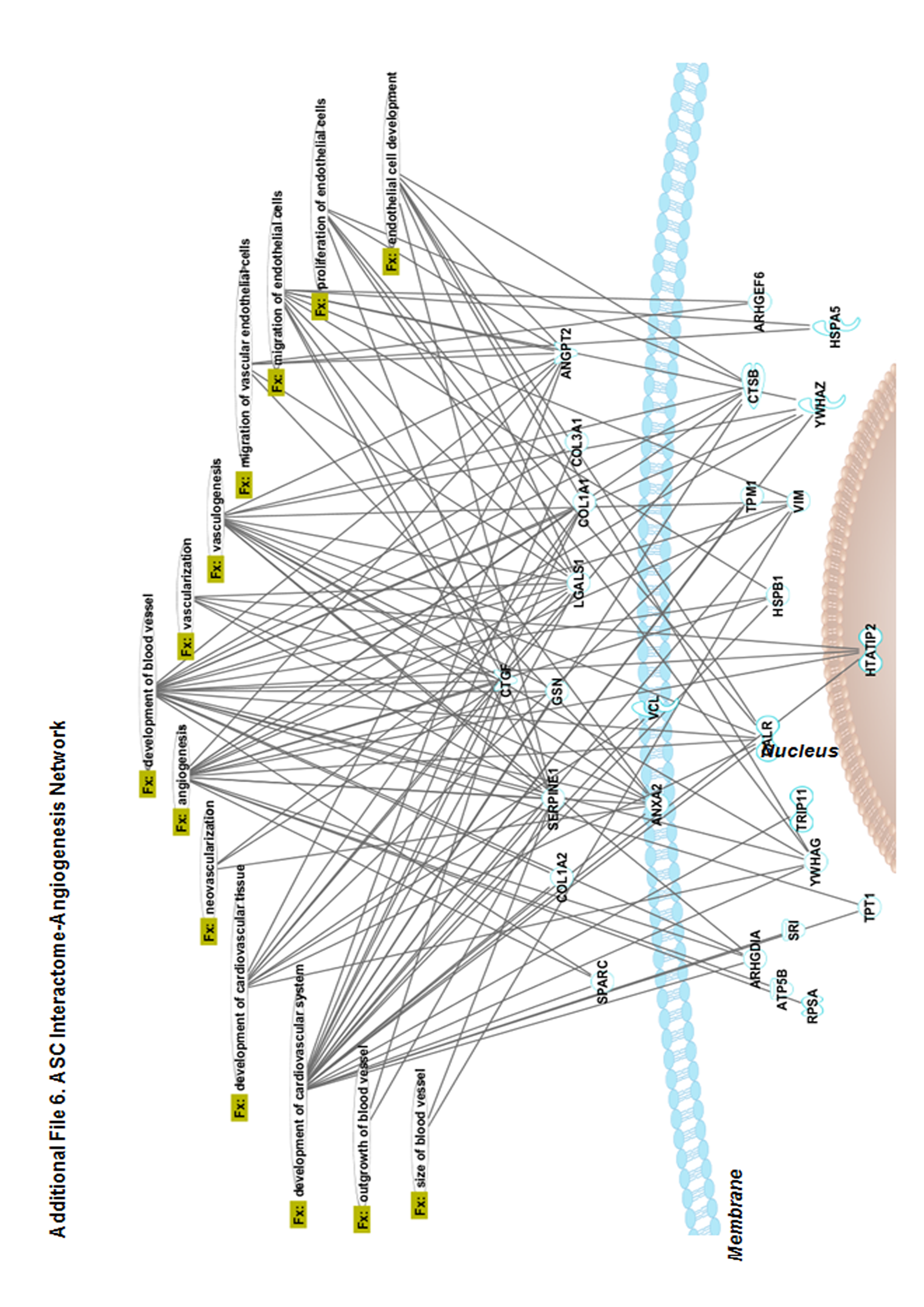

Supplement: Additional file 6: — Is a figure showing the ASC interactome–angiogenesis network. (TIF 11036 kb) [file 13287_2017_509_MOESM6_ESM.tif]
