# Supplementary material for: Allogenic adipose-derived stem cell therapy overcomes ischemia-induced microvessel rarefaction in the myocardium: systems biology study
Source: Stem Cell Res Ther. 2017 Mar 9;8:52. doi: 10.1186/s13287-017-0509-2 (PMC5345145; doi:10.1186/s13287-017-0509-2)
Supplement: Additional file 5: — Is Table S1 presenting identified proteins by MALDI-TOF/TOF in ASCs and CM (ASC releasate). (DOC 171 kb) [file 13287_2017_509_MOESM5_ESM.doc]

**Table S1. Identified proteins by MALDI-TOF/TOF in ASC and CM (ASC-releasate).**

| **Spot No** | **ASC** | **CM** | **ProtName** | **Mascot Score** | **Sequence coverage** | **MW** | **pI** | **SwissProt Name** | **SwissProt No** | **Functional group** |
| --- | --- | --- | --- | --- | --- | --- | --- | --- | --- | --- |
| 1 |  |  | Angiopoietin-2 | 51 | 14 | 56.99 | 6.4 | ANGP2 | [O15123](http://www.uniprot.org/uniprot/O15123) | Angiogenesis |
| 2 |  |  | Programmed cell death protein 6 | 78 | 24 | 21.91 | 5.0 | PDCD6 | O75340 | Angiogenesis |
| 3 |  |  | Plasminogen activator inhibitor 1 | 44 | 2* | 45.48 | 6.9 | PAI1 | P05121 | Angiogenesis |
| 4 |  |  | ATP synthase subunit beta, mitochondrial | 174 | 40 | 56.53 | 5.1 | ATP5B | P06576 | Angiogenesis |
| 5 |  |  | Annexin A2 | 58 | 20 | 38.92 | 7.6 | ANXA2 | P07355 | Angiogenesis |
| 6 |  |  | Connective tissue growth factor | 37 | 3* | 40.26 | 10 | CTGF | P29279 | Angiogenesis |
| 7 |  |  | Oxidoreductase HTATIP2 | 78 | 37 | 27.23 | 9.4 | HTAI2 | Q9BUP3 | Angiogenesis |
| 8 |  |  | Thioredoxin | 68 | 31 | 12.01 | 4.6 | THIO | P10599 | Antioxidant/Redox Homeostasis |
| 9 |  |  | Peroxiredoxin-6 | 80 | 33 | 24.86 | 5.6 | PRDX6 | P30041 | Antioxidant/Redox Homeostasis |
| 10 |  |  | Peroxiredoxin-4 | 55 | 19 | 30.75 | 5.8 | PRDX4 | Q13162 | Antioxidant/Redox Homeostasis |
| 11 |  |  | SPARC | 65 | 4* | 35.45 | 4.6 | SPRC | P09486 | Cell proliferation/Differentiation/Apoptosis regulation |
| 12 |  |  | Rho GDP-dissociation inhibitor 1 | 102 | 47 | 23.46 | 5.0 | GDIR1 | P52565 | Cell proliferation/Differentiation/Apoptosis regulation |
| 13 |  |  | Peroxiredoxin-1 | 48 | 5* | 22.32 | 9.2 | PRDX1 | [Q06830](http://www.uniprot.org/uniprot/Q06830) | Cell proliferation/Differentiation/Apoptosis regulation |
| 14 |  |  | Annexin A1 | 64 | 4* | 39.21 | 6.4 | ANXA1 | P04083 | Cell proliferation/Differentiation/Apoptosis regulation |
| 15 |  |  | Alpha-enolase | 117 | 30 | 47.64 | 6.4 | ENO1 | P06733 | Cell proliferation/Differentiation/Apoptosis regulation |
| 16 |  |  | Annexin A5 | 160 | 47 | 35.97 | 4.8 | ANXA5 | P08758 | Cell proliferation/Differentiation/Apoptosis regulation |
| 17 |  |  | Galectin-1 | 109 | 11 | 15.03 | 4.8 | LGALS1 | P09382 | Cell proliferation/Differentiation/Apoptosis regulation |
| 18 |  |  | Calreticulin | 82 | 11 | 12.01 | 5.5 | CALR | P27797 | Cell proliferation/Differentiation/Apoptosis regulation |
| 19 |  |  | 14-3-3 protein gamma | 118 | 40 | 28.46 | 4.7 | YWHAG | P61981 | Cell proliferation/Differentiation/Apoptosis regulation |
| 20 |  |  | Dihydropyrimidinase-related protein 2 | 158 | 31 | 62.64 | 5.9 | DPYSL2 | Q16555 | Cell proliferation/Differentiation/Apoptosis regulation |
| 21 |  |  | Interferon kappa | 52 | 20 | 23.85 | 10 | IFNK | Q9P0W0 | Cell proliferation/Differentiation/Apoptosis regulation |
| 22 |  |  | Annexin A8 | 113 | 4* | 36.99 | 5.2 | ANXA8 | P13928 | Coagulation/Haemostasis |
| 23 |  |  | Coactosin-like protein | 66 | 33 | 16.05 | 5.4 | COLT1 | Q14019 | Defense response |
| 24 |  |  | Glycogen phosphorylase, liver form | 63 | 16 | 97.81 | 6.3 | PYGL | P06737 | Metabolism |
| 25 |  |  | L-lactate dehydrogenase B chain | 76 | 20 | 36.87 | 5.5 | LDHB | P07195 | Metabolism |
| 26 |  |  | Proteasome subunit alpha type-5 | 39 | 4* | 26.57 | 4.6 | PSA5 | P28074 | Protein translation/Folding/Chaperone |
| 27 |  |  | Heat shock protein beta-1 (HSP27) | 81 | 39 | 22.44 | 6.0 | HSPB1 | P04792 | Protein translation/Folding/Chaperone |
| 28 |  |  | Protein disulfide-isomerase | 119 | 3* | 57.63 | 4.7 | PDIA1 | P07237 | Protein translation/Folding/Chaperone |
| 29 |  |  | Heat shock 70 kDa protein 1A/1B | 192 | 25 | 70.29 | 5.4 | HSPA1A | P08107 | Protein translation/Folding/Chaperone |
| 30 |  |  | 40S ribosomal protein SA (Laminin receptor 1) | 120 | 36 | 32.98 | 4.7 | LAMR1 | P08865 | Protein translation/Folding/Chaperone |
| 31 |  |  | Ubiquitin carboxyl-terminal hydrolase isozyme L1 | 81 | 38 | 25.19 | 5.1 | UCHL1 | P09936 | Protein translation/Folding/Chaperone |
| 32 |  |  | 60 kDa heat shock protein, mitochondrial | 106 | 2* | 61.12 | 5.7 | HSPD1 | P10809 | Protein translation/Folding/Chaperone |
| 33 |  |  | 78 kDa glucose-regulated protein | 156 | 22 | 72.40 | 4.9 | HSPA5 | P11021 | Protein translation/Folding/Chaperone |
| 34 |  |  | Heat shock cognate 71 kDa protein | 203 | 28 | 70.99 | 5.1 | HSPA8 | P11142 | Protein translation/Folding/Chaperone |
| 35 |  |  | Endoplasmin (GRP 94) | 73 | 7* | 92.74 | 4.6 | HSP90B1 | P14625 | Protein translation/Folding/Chaperone |
| 36 |  |  | Adenosylhomocysteinase | 82 | 25 | 48.18 | 5.9 | AHCY | P23526 | Protein translation/Folding/Chaperone |
| 37 |  |  | Proteasome subunit alpha type-6 | 77 | 24 | 27.84 | 6.4 | PSMB6 | P28072 | Protein translation/Folding/Chaperone |
| 38 |  |  | Protein disulfide-isomerase A3 (GRP58) | 114 | 34 | 57.15 | 5.9 | PDIA3 | P30101 | Protein translation/Folding/Chaperone |
| 39 |  |  | Mortalin (GRP75) | 123 | 2* | 73.98 | 5.9 | GRP75 | P38646 | Protein translation/Folding/Chaperone |
| 40 |  |  | Eukaryotic initiation factor 4A-I | 91 | 30 | 45.49 | 5.2 | IF4A1 | P60842 | Protein translation/Folding/Chaperone |
| 41 |  |  | GRIP and coiled-coil domain-containing protein 2 | 69 | 6* | 185.51 | 4.9 | GCC2 | Q8IWJ2 | Protein translation/Folding/Chaperone |
| 42 |  |  | Sacsin | 72 | 7* | 526.50 | 6.7 | SACS | Q9NZJ4 | Protein translation/Folding/Chaperone |
| 43 |  |  | Cathepsin D | 62 | 14 | 37.73 | 6.4 | CTSD | P07339 | Proteolysis |
| 44 |  |  | Cathepsin B | 66 | 17 | 37.90 | 5.8 | CTSB | P07858 | Proteolysis |
| 45 |  |  | 14-3-3 protein epsilon | 134 | 52 | 29.33 | 4.5 | YWHAE | B4DJF2 | Signaling/Gene Transcription |
| 46 |  |  | Chloride intracellular channel protein 1 | 95 | 29 | 27.32 | 5.0 | CLIC1 | O00299 | Signaling/Gene Transcription |
| 47 |  |  | Annexin A6 | 71 | 11 | 76.17 | 5.3 | ANXA6 | P08133 | Signaling/Gene Transcription |
| 48 |  |  | Nucleoside diphosphate kinase A | 75 | 39 | 17.30 | 5.9 | NME1 | P15531 | Signaling/Gene Transcription |
| 49 |  |  | Protein S100-A4 | 91 | 43 | 11.95 | 5.8 | S100A4 | P26447 | Signaling/Gene Transcription |
| 50 |  |  | Rho guanine nucleotide exchange factor 6 | 60 | 9* | 88.70 | 5.7 | ARHGEF6 | Q15052 | Signaling/Gene Transcription |
| 51 |  |  | Nucleoside-triphosphatase C1orf57 | 58 | 28 | 20.93 | 10.5 | NTPCR | Q9BSD7 | Signaling/Gene Transcription |
| 52 |  |  | Zinc finger protein 101 | 64 | 19 | 51.90 | 10.8 | ZN101 | Q8IZC7 | Signaling/Gene Transcription |
| 53 |  |  | 14-3-3 protein zeta/delta | 128 | 44 | 27.90 | 4.6 | YWHAZ | P63104 | Signaling/Gene Transcription |
| 54 |  |  | 14-3-3 protein theta | 57 | 18 | 28.03 | 4.5 | YWHAQ | P68254 | Signaling/Gene Transcription |
| 55 |  |  | Collagen alpha-1(I) chain | 90 | 0* | 139.88 | 5.5 | CO1A1 | P02452 | Structural |
| 56 |  |  | Collagen alpha-1(III) chain | 98 | 1* | 139.73 | 6.2 | CO3A1 | P02461 | Structural |
| 57 |  |  | Collagen alpha-2(I) chain | 53 | 0* | 129.50 | 9.9 | CO1A2 | P08123 | Structural |
| 58 |  |  | Fibrosin-1 | 64 | 25 | 19.93 | 11.8 | FBS1 | [Q9HAH7](http://www.uniprot.org/uniprot/Q9HAH7) | Structural |
| 59 |  |  | Integrin alpha-11 | 61 | 5 | 134.13 | 6.4 | ITA11 | [Q9UKX5](http://www.uniprot.org/uniprot/Q9UKX5) | Structural |
| 60 |  |  | Smoothelin-like protein 1 | 63 | 26 | 49.21 | 4.5 | SMTL1 | A8MU46 | Structural |
| 61 |  |  | Gelsolin | 54 | 13 | 85.07 | 5.9 | GELS | P06396 | Structural |
| 62 |  |  | Tropomyosin alpha-3 chain | 137 | 44 | 29.22 | 4.6 | TPM3 | P06753 | Structural |
| 63 |  |  | Tubulin beta chain (Tubulin beta-5 chain) | 68 | 3 | 50.29 | 4.6 | TBB | P07437 | Structural |
| 64 |  |  | Tropomyosin beta chain (Tropomyosin-2) | 65 | 37 | 32.93 | 4.5 | TPM2 | P07951 | Structural |
| 65 |  |  | Vimentin | 209 | 57 | 53.68 | 4.9 | VIME | P08670 | Structural |
| 66 |  |  | Tropomyosin alpha-1 chain (Tropomyosin-1) | 103 | 7* | 32.73 | 4.5 | TPM1 | P09493 | Structural |
| 67 |  |  | Plastin-2 | 26 | 2* | 70.82 | 5.1 | LCP1 | P13796 | Structural |
| 68 |  |  | Myosin regulatory light chain RLC-A | 66 | 5* | 19.94 | 4.5 | MRLCA | P13832† | Structural |
| 69 |  |  | Vinculin | 111 | 13 | 124.44 | 5.5 | VCL | P18206 | Structural |
| 70 |  |  | Myosin regulatory light polypeptide 9 | 53 | 5* | 19.87 | 4.6 | MYL9 | P24844 | Structural |
| 71 |  |  | Macrophage-capping protein | 56 | 4* | 38.78 | 5.9 | CAPG | P40121 | Structural |
| 72 |  |  | Actin, cytoplasmic 1 (Beta-actin) | 110 | 29 | 40.76 | 5.5 | ACTB | P60709 | Structural |
| 73 |  |  | Actin-related protein 3 | 128 | 27 | 47.80 | 5.5 | ACTR3 | P61158 | Structural |
| 74 |  |  | Actin, aortic smooth muscle | 145 | 4* | 42.38 | 5.1 | ACTA | P62736 | Structural |
| 75 |  |  | Tropomyosin alpha-4 chain (Tropomyosin-4) | 118 | 49 | 28.62 | 4.5 | TPM4 | P67936 | Structural |
| 76 |  |  | Fascin (55 kDa actin-bundling protein) | 59 | 12 | 55.12 | 7.0 | FSCN1 | Q16658 | Structural |
| 77 |  |  | Tubulin alpha-1C chain (Tubulin alpha-6 chain) | 98 | 27 | 50.56 | 4.8 | TBA1C | Q9BQE3 | Structural |
| 78 |  |  | Talin-1 | 62 | 4* | 271.83 | 5.8 | TLN1 | Q9Y490 | Structural |
| 79 |  |  | Ferritin light chain | 119 | 19 | 8.84 | 5.8 | FTL | P02792 | Trasport/Trafficking |
| 80 |  |  | Translationally-controlled tumor protein (Fortilin) | 34 | 5* | 19.68 | 4.7 | TCTP | P13693 | Trasport/Trafficking |
| 81 |  |  | Sorcin | 34 | 6* | 21.95 | 5.2 | SORCN | P30626 | Trasport/Trafficking |
| 82 |  |  | Coatomer subunit delta | 75 | 21 | 57.62 | 5.8 | COPD | P48444 | Trasport/Trafficking |
| 83 |  |  | Rab GDP dissociation inhibitor beta | 178 | 40 | 50.75 | 6.3 | GDI2 | P50395 | Trasport/Trafficking |
| 84 |  |  | Thyroid receptor-interacting protein 11 | 70 | 5* | 228.18 | 5.1 | TRIP11 | Q15643 | Trasport/Trafficking |
